# Clinical relevance of CERK and SPHK1 in breast cancer and their association with metastasis and drug resistance

**DOI:** 10.1038/s41598-022-20976-0

**Published:** 2022-10-29

**Authors:** Priyanka Bhadwal, Vinay Randhawa, Kim Vaiphei, Divya Dahiya, Navneet Agnihotri

**Affiliations:** 1 Department of Biochemistry, Panjab University, Chandigarh, India; 2 Cardiovascular Division, Department of Medicine, Brigham and Women’s Hospital, Harvard Medical School, Boston, MA USA; 3 Department of Histopathology, PGIMER, Chandigarh, India; 4 Department of General Surgery, PGIMER, Chandigarh, India

**Keywords:** Cancer, Biomarkers

## Abstract

Despite numerous reports on the altered sphingolipids metabolism in human cancers, their clinical significance in breast cancer remains obscure. Previously, we identified the high levels of sphingolipids, ceramide phosphates and sphingosine phosphates, and the genes involved in their synthesis, *CERK* and *SPHK1*, in breast cancer patients. The present study aimed to determine the correlations of *CERK* and *SPHK1* with clinical outcomes as well as metastasis and drug resistance markers. Both local and TCGA cohorts were analysed. High-confidence regulatory interaction network was constructed to find association of target genes with metastasis and drug resistance. Furthermore, correlations of *CERK* and *SPHK1* with selected metastasis and drug resistance markers were validated in both cohorts. Overexpression of *CERK* and *SPHK1* was associated with nodal metastasis, late tumor stage and high proliferation potency. In addition, increased *CERK* expression was also indicative of poor patient survival. Computational network analysis revealed the association of *CERK* and *SPHK1* with known metastasis markers *MMP-2* and *MMP-9* and drug resistance markers *ABCC1* and *ABCG2*. Correlation analysis confirmed the associations of target genes with these markers in both local as well as TCGA cohort. The above findings suggest clinical utility of *CERK* and *SPHK1* as potential biomarkers in breast cancer patients and thus could provide novel leads in the development of therapeutics.

## Introduction

Breast cancer is the most common neoplasm in women and is the leading cause of morbidity and mortality worldwide^1^. In 2020, approximately 22,61,419 new cases of breast cancer were reported globally^2^. Studies found that the chances of a woman to be diagnosed with breast cancer during her lifetime is approximately 1 in 8 women (12.5%) while the chances of dying from the disease is 1 in 39 women (2.5%)^3^. Accumulating evidence suggest that early-stage screening and detection of the cancer can lead to improved diagnosis and prognosis and reduce the associated mortality. Despite the availability of various screening and treatment modalities, the incidence rate of breast cancer is increasing at an alarming rate. To overcome the existing lacunae, current studies are aligned towards the development of reliable biomarkers that can quantify the risk of patients and favour a better therapeutic approach early in the course of treatment.

A major clinical challenge associated with successful breast cancer therapy is metastasis. Metastatic tumors in most cases are reported to be resistant to cytotoxic drugs and other conventional treatments. Distant metastatic breast cancer (MBC) is the most severe form of breast cancer and the five-year survival rate of MBC is only 27%^3^. Tumor cells interact with their microenvironment and elicit the expression of growth factors, chemokines, matrix metalloproteinases (MMPs) which lead to metastasis by inducing the process of epithelial mesenchymal transition (EMT)^4^. In addition to metastasis, multidrug resistance (MDR) in tumor cells is another crucial obstacle that impairs the efficacy of both conventional and novel molecular therapies. The principal molecular mechanism involved in drug resistance is the increased rate of drug efflux by the members of the ATP-Binding Cassette (ABC) transporter superfamily such as ABCB1, ABCC1 and ABCG2^5^. Overexpression of these drug transporters has been found to be associated with MDR in prostate, small cell lung carcinoma and breast cancer^6^. In addition to their role in drug efflux, ABC transporters also mediate the movement of lipids and other metabolic products across the plasma and intracellular membranes.

Sphingolipids are an important class of lipids that play key cellular roles both as structural components of membranes as well as signaling molecules. The metabolism of sphingolipids generates bioactive metabolites that participate in the regulation of cancer development, proliferation, progression, metastasis and chemoresistance in various cancer types^7^. The sphingolipid metabolite, sphingosine-1-phosphate (S1P) is known to contribute to EMT pathway by modulating the levels of MMPs such as MMP-2 and MMP-9^8^. Increased expression of *SPHK1* has also been shown to promote colon cancer growth and progression by inducing the expression of MMP-2 and MMP-9^9,10^. Numerous studies have linked the development of drug resistance to changes in the sphingolipid metabolism^11–13^.

Our group has previously identified sphingolipid metabolites ceramide-1-phosphate (CerP), S1P and sphingomyelins (SM) to be differentially regulated between tumor and adjacent normal breast tissues and their clinical relevance as biomarkers was determined^14^. Consistent with the above results, an upregulation in the level of genes, ceramide kinase (*CERK*), sphingosine kinase 1 (*SPHK1*) and sphingomyelin synthase 1 (*SGMS1*), involved in the synthesis of these metabolites was also observed in local and The Cancer Genome Atlas (TCGA) cohort. Of the abovementioned genes, *CERK* and *SPHK1* had a significant potential to discriminate between tumor and adjacent normal breast tissues. However, the clinical utility of these genes and their association with cancer cellular processes in breast cancer is still not explored and needs further understanding.

In the present study, we analysed the clinicopathological correlations of *CERK* and *SPHK1*, and their ability to predict patient outcome in various clinical sub-groups. Furthermore, interaction network-based analyses were performed (by integrating gene co-expression regulations and physical PPIs) to determine the functional relatedness of these genes with metastasis and drug resistance in breast cancer. The findings were then validated in local as well as TCGA cohort.

## Results

The clinicopathological characteristics of the patients were described in our previous study^14^.

### Histopathological examinations of breast tissue

**Figure 1:**
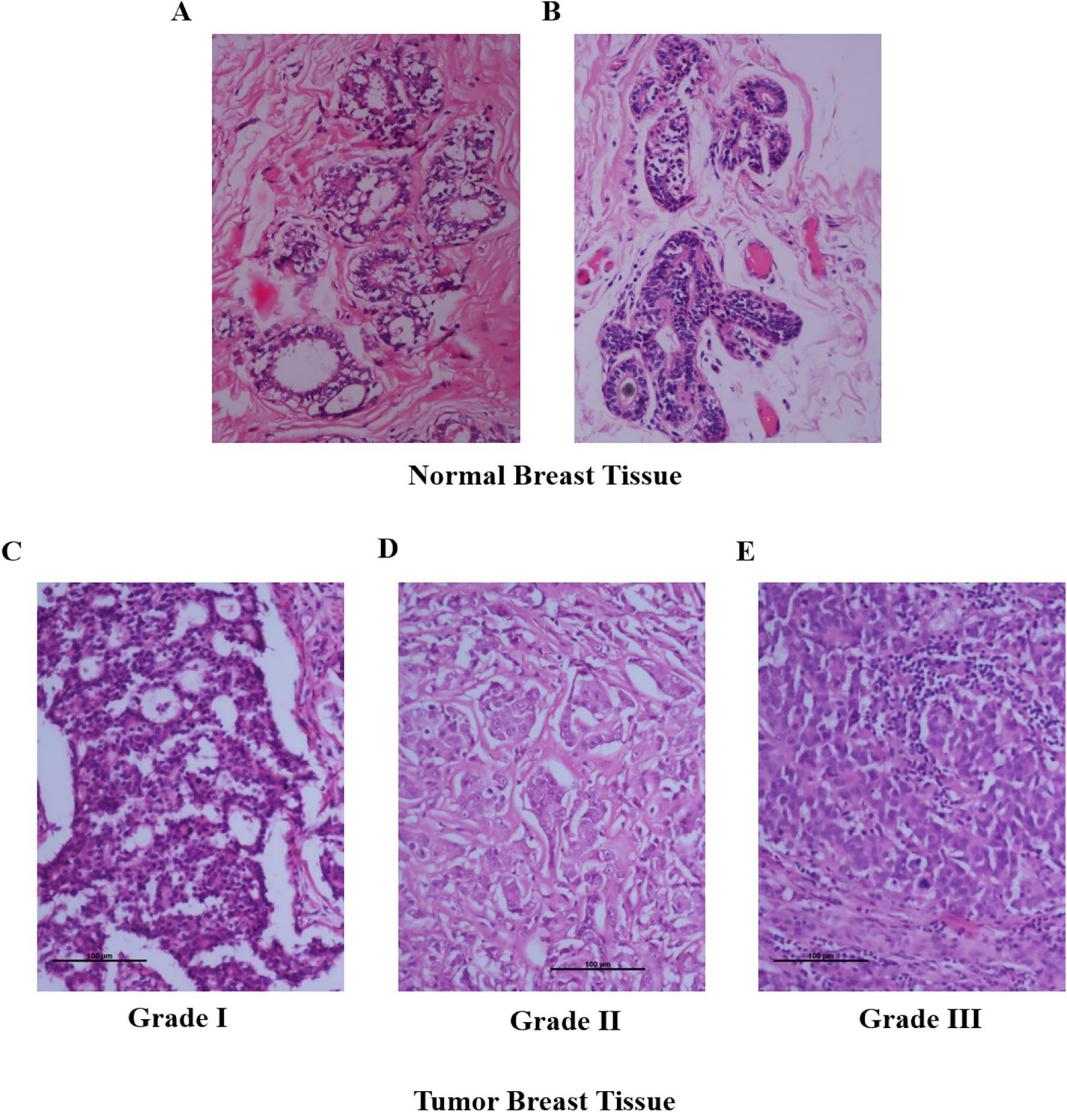
Representative images of H & E-stained sections of breast tissue. (**A** and **B**) Normal Breast Tissue, (**C**–**E**) Tumor Breast Tissue, (**C**) Grade I, (**D**) Grade II, (**E**) Grade III. Bar represents 100 μm on the image.

Morphological assessment of breast biopsy tissue was carried out on routine hematoxylin & eosin (H & E) -stained paraffin sections. Normal breast epithelial cells in the terminal duct lobular units had uniform shapes and sizes having uniform monomorphic nuclei with finely dispersed chromatin (Fig. 1A and B). Tumour cells were arranged in tubules, clusters, cords and trabeculae. Tumour cell with grade I histology showed mildly enlarged nuclei which are fairly uniform with visible nucleoli, mitosis was infrequent (Fig. 1C). Grade II tumour cells showed moderate degree nuclear pleomorphism, moderately enlarged and occasionally showing tubular differentiation. Mitosis was occasionally visible (Fig. 1D). Tumour cells in a high grade or grade III carcinoma showed gross nuclear pleomorphism arranged in sheets, large clusters and trabeculae, no tubular differentiation was visible. Individual cells showed marked nuclear pleomorphism, irregularly clumped chromatin, prominent nucleoli and frequent mitosis. (Fig. 1E).

### Clinical Relevance of *CERK* and *SPHK1*

#### Correlation of *CERK *and *SPHK1* with clinicopathological characteristics

**Figure 2:**
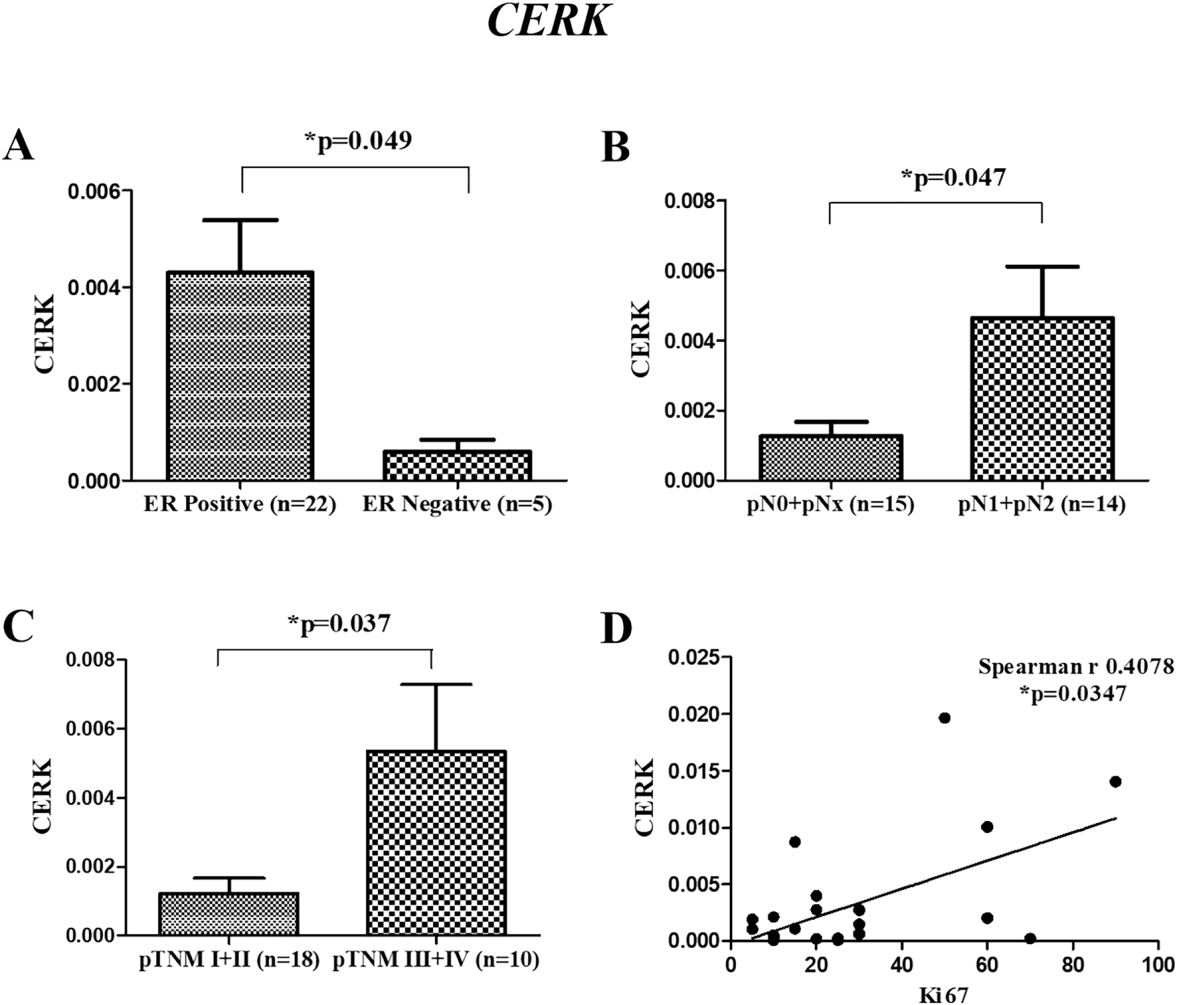
Correlation of *CERK* gene expression with various clinicopathological characteristics in breast tumor tissues. (**A**) ER Status (**B**) pN Stage (pN0 + pNx- [Nodal Negative], pN1 + pN2- [Nodal Positive]) (**C**) pTNM Stage (pTNM I + II- [early stage], pTNM III + IV- [late stage]) (**D**) Correlation with Ki67.

**Figure 3:**
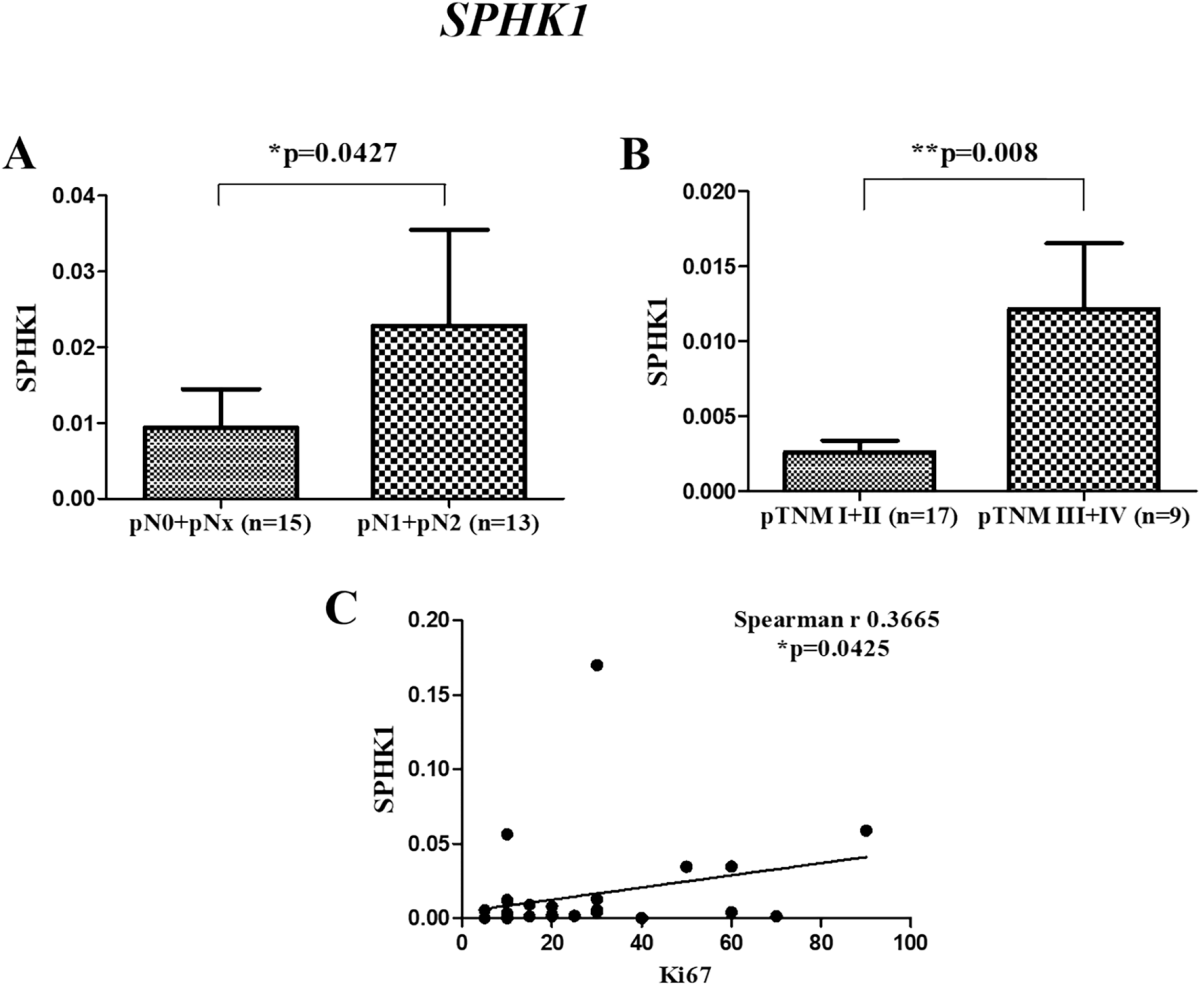
Correlation of *SPHK1* gene expression with various clinicopathological characteristics in breast tumor tissues. (**A**) pN Stage (pN0 + pNx- [Nodal Negative], pN1 + pN2-[Nodal Positive]) (**B**) pTNM Stage (pTNM I + II- [early stage], pTNM III + IV- [late stage]) (**C**) Correlation with Ki67.

We previously reported an overexpression of *CERK* and *SPHK1* in breast tumor tissues as compared to adjacent normal tissues^14^. To further strengthen the clinical relevance of these genes in breast cancer patients, their correlations with age, ER status, tumor grade, nodal status, tumor stage and proliferation marker were evaluated. There was no significant difference observed in the expression of these genes according to age and tumor grades of patients. Significant high level of *CERK* was observed in ER Positive (*p = 0.049) (Fig. 2A), nodal positive (*p = 0.047) (Fig. 2B) and late-stage breast cancer patients (*p = 0.037) (Fig. 2C). A significant positive correlation was also observed between the levels of *CERK* and proliferation marker Ki67 (*p = 0.0347) (Fig. 2 D). Similar results were observed for *SPHK1,* with high levels in nodal positive patients (*p = 0.0427) (Fig. 3A), late-stage patients (**p = 0.008) (Fig. 3B), and a positive association with Ki67 (*p = 0.0425) (Fig. 3C). The expression of *SPHK1* was not observed to be corelated with the ER status of the patient. In TCGA cohort, no significant correlation of *CERK* and *SPHK1* with clinicopathological characteristics was observed.

#### Protein expression of *CERK* and *SPHK1* in breast cancer patients

**Figure 4:**
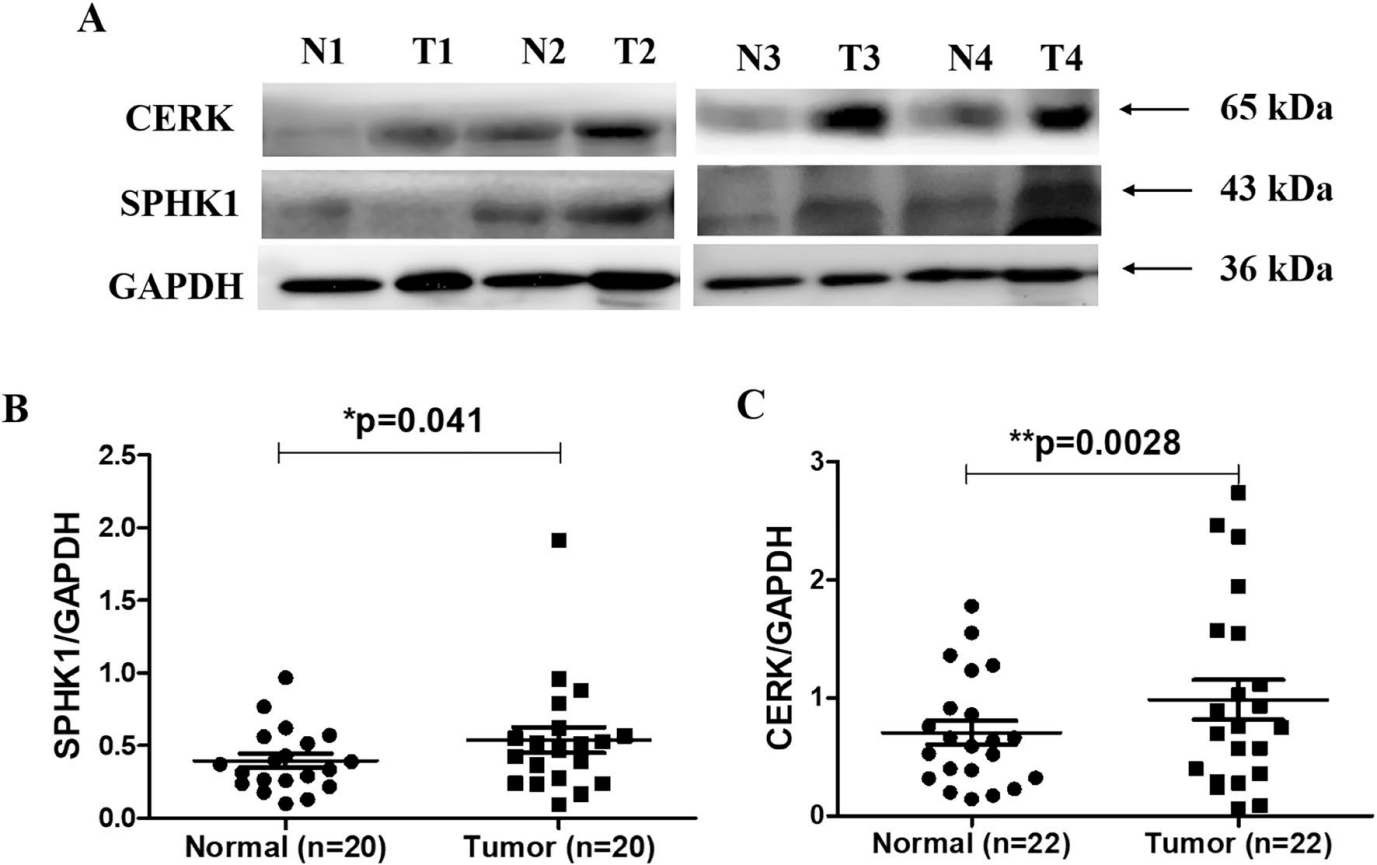
Protein expression of SPHK1 and CERK in breast cancer patients. (**A**) Representative blots in adjacent normal (N) and tumor (T) tissues, (**B**) Densitometric analysis of SPHK1 and (**C**) CERK levels in adjacent normal and tumor tissues.

To further validate alterations in the levels of *CERK and SPHK1*, the levels of corresponding proteins were also analysed in local cohort samples using western blotting (Fig. 4A) (Supplementary Figs. 1 and 4). In the present study, protein expression of both SPHK1 (*p = 0.041) (Fig. 4 B) and CERK (**p = 0.0028) (Fig. 4C) were found to be significantly upregulated in tumor tissue as compared to adjacent normal tissue.

#### Correlation of *CERK *and *SPHK1* with overall survival (OS) in breast cancer

**Figure 5:**
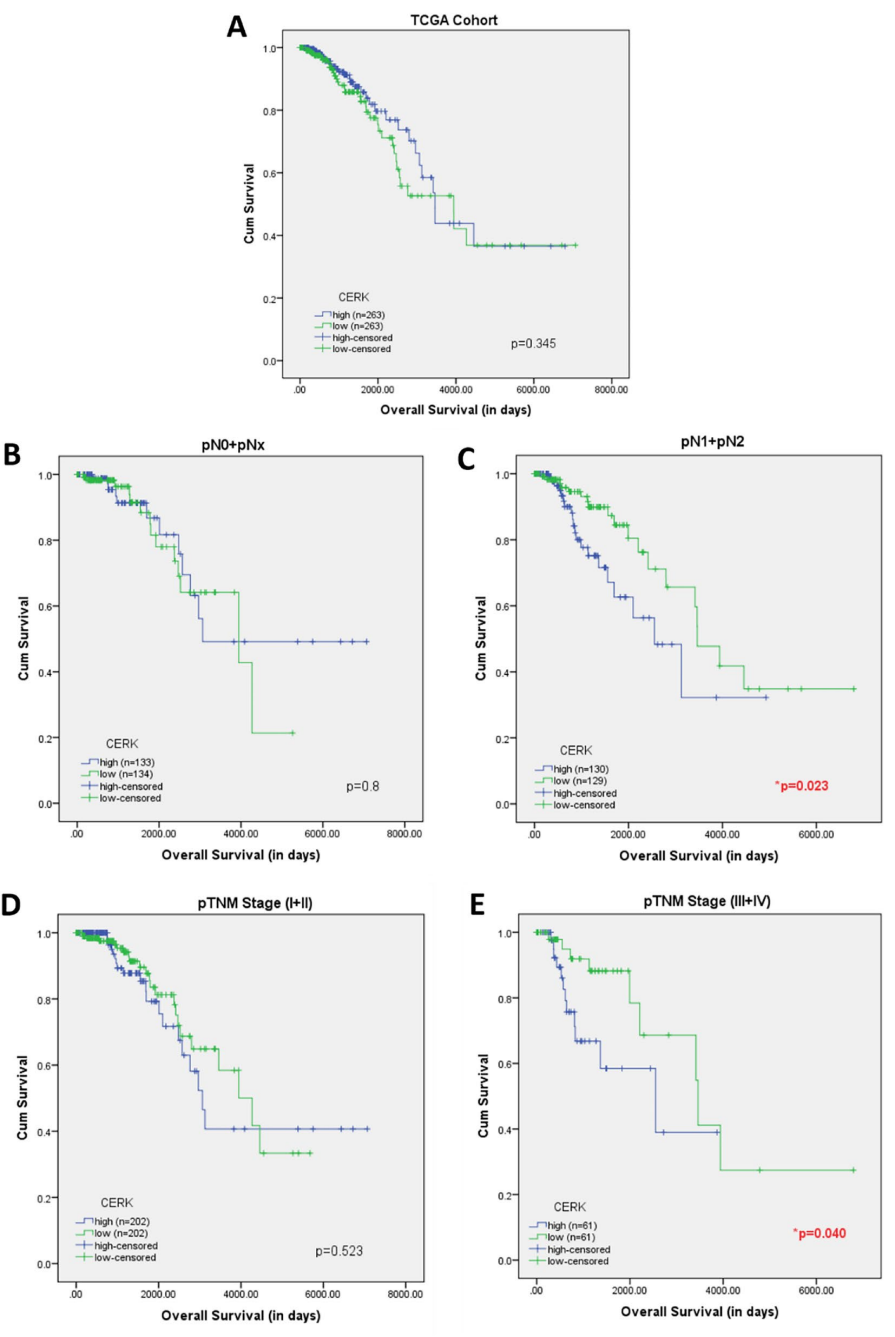
(**A**) Kaplan Meier survival curves showing OS in TCGA cohort stratified by CERK expression, (**B**) pN0 + pNx [Nodal Negative], (**C**) pN1 + pN2 [Nodal Positive], (**D**) pTNM (I + II)-[Early Stage], (**E**) pTNM (III + IV) [Late Stage]*.*

To test the prognostic values of *CERK* and *SPHK1*, their association with OS was studied in breast cancer patients using Kaplan–Meier plot. A total of 526 breast cancer patients from TCGA dataset were divided into low expression and high expression group based on the median expression. There was no association found between the expression of *CERK* and OS (Fig. 5A). Similarly, *SPHK1* was also not found to correlate with the OS of breast cancer patients. To further understand the relationship between the genes and survival, the patients were divided into various subgroups. The expression of *SPHK1* was observed to have no impact on OS among various breast cancer subgroups. However, high *CERK* expression was related to poor OS in patients with lymph node metastasis (*p = 0.023) (Fig. 5C) and late-stage breast cancer patients (*p = 0.040) (Fig. 5E). No other associations were observed between *CERK* expression and other clinical subgroups (Fig. 5B and D).

### Relationship of *CERK* and *SPHK1* with metastasis and drug resistance

#### Network inference and its characteristics

**Figure 6:**
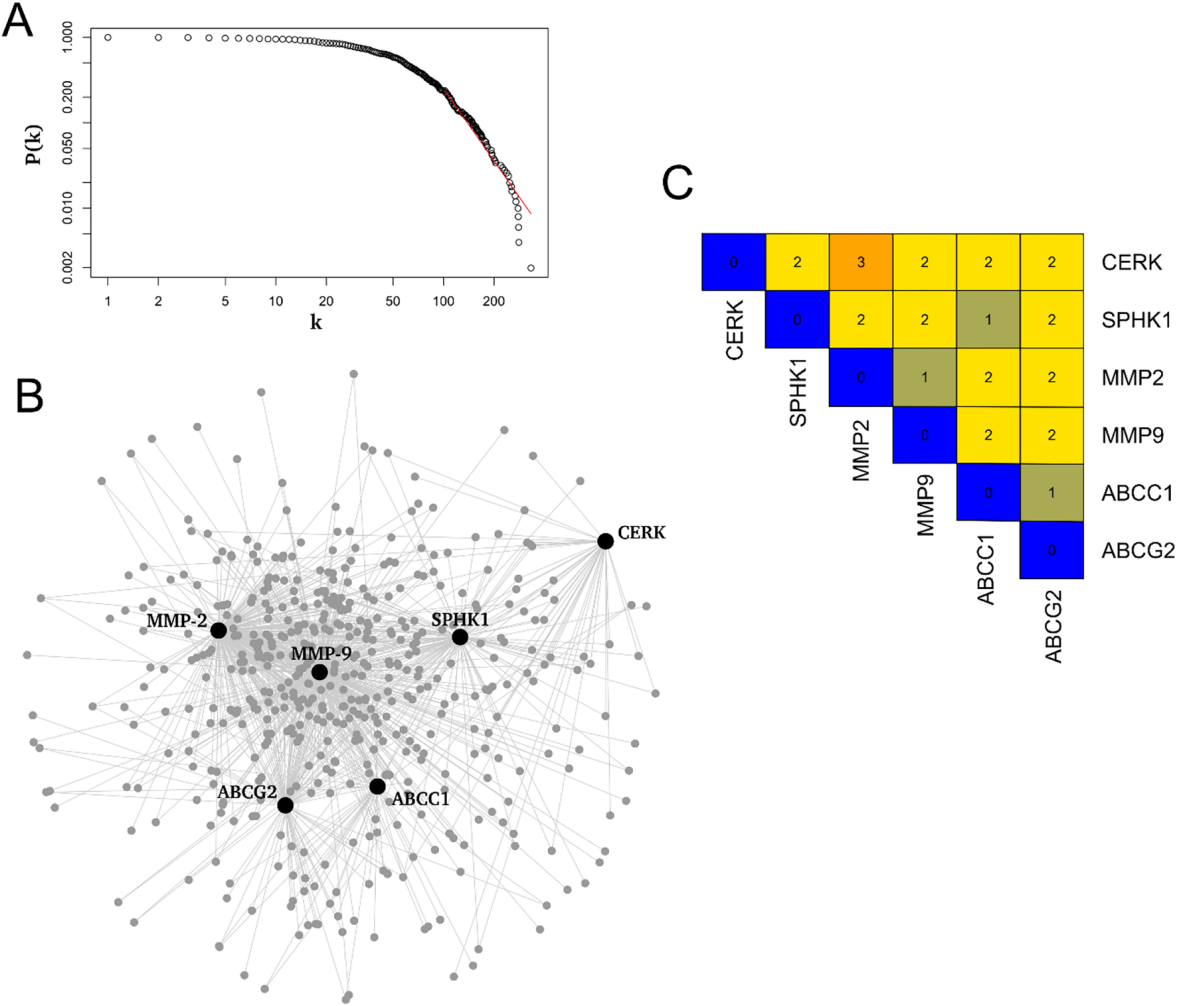
Network-based analysis of interaction network corresponding to candidate genes. (**A**) The node degree distribution of PPI network. The number of genes is plotted as a function of their degree reflecting a power-law like distribution. The red line corresponds to a power-law distribution. (**B**) Sub-network of candidate genes. The large-sized black-colored nodes represent the candidate genes (*ABCC1*, *ABCG2*, *CERK*, *MMP-2*, *MMP-9*, and *SPHK1*), while the small gray color nodes represent the corresponding interactor genes. Interactions are represented in gray color and the depth of color represents the strength of correlations. (**C**) Shortest path lengths among candidate genes. Heatmap of shortest path length among the candidate genes, where the values represent the number of shortest paths between any pair.

To obtain an understanding of regulatory interactions between *CERK*, *SPHK1*, and metastasis and drug resistance markers, the underlying protein–protein interaction (PPI) network was inferred. The PPI network comprised 18,066 interactions among 506 proteins, including candidate proteins (*CERK* and *SPHK1*) along with metastasis markers, *MMP-2* and *MMP-9*, and drug resistance markers, *ABCC1* and *ABCG2*. To affirm the biological significance of network, the degree distribution of established PPI network was computed and compared to randomly generated networks. The PPI network was highly skewed and the node degree distribution approximated the power-law distribution (Fig. 6A), indicating the biological significance of network and presence of some exceptionally connected proteins. In this aspect, topological analyses of the network were performed to assess the centrality of candidate proteins. Centrality measure k specifies the number of interactions a protein has within the interaction network and the degree distribution predict the highly connected proteins called hubs^15,16^. The other centrality measure betweenness centrality (BC) measures the information flow across the network, and predicts non-hubs that play roles of bottlenecks in the network^17^. Both k and BC centrality measures are well-known to be important in selecting globally and locally important proteins respectively, in the network^18^ and were therefore considered for the network analysis after assessing their biological relevance (Supplementary Data R1). Metastasis markers *MMP-2* and *MMP-9* were among the top-hub as well as among the top-bottlenecks whereas *ABCC1* and *ABCG2* were only among the top-bottleneck drug resistance proteins. While *ABCC1* and *ABCG2* drug transporters were the primary interactors of target proteins, another transporter, Spinster homolog 2 (SPNS2) was also observed in the network as a transitive protein between *CERK* and *ABCG2*. *CERK* was neither a network hub nor a bottleneck. While *SPHK1* was not a network hub, it was among the top-bottleneck proteins indicating it be a highly ‘between’ among small network clusters and thus could possibly play important roles in mediating signaling during cancer progression.

To explore the network-based correlations among *CERK* and *SPHK1*, *MMP-2* and *MMP-9*, and *ABCC1* and *ABCG2*, a sub-network comprising interactions among candidates was extracted (Fig. 6B) from the large PPI network. In biological aspects, shortest path lengths compute the ‘functional closeness’ of proteins in a biological network. *CERK* and *SPHK1* had smaller shortest paths across each other as well as across metastasis and drug resistance markers, and the length were nearly uniform (Fig. 6C). *SPHK1* had shortest paths of 2 across *MMP-2*, *MMP-9*, and *ABCG2*, whereas shortest path of 1 with *ABCC1*. Similarly, *CERK* also had shortest path length of 2 across *MMP-2*, *MMP-9*, *ABCG2*, and *ABCC1*. *CERK* was functionally close with other candidates*,* indicating it to be playing major role in maintaining the regulatory interactions during breast cancer. All these findings indicate the functional closeness of *CERK* and *SPHK1* with metastasis and drug resistance markers and thus could regulate them through signaling pathways. Identification of smaller shortest paths between candidate genes also determined various transitive proteins (Supplementary Table S1) acting as important intermediates regulating the signaling during cancerous conditions.

#### Expression of MMP-2, MMP-9, ABCC1, and ABCG2 and their correlation with target genes

##### Analysis of gene and protein expression of metastasis markers

**Figure 7:**
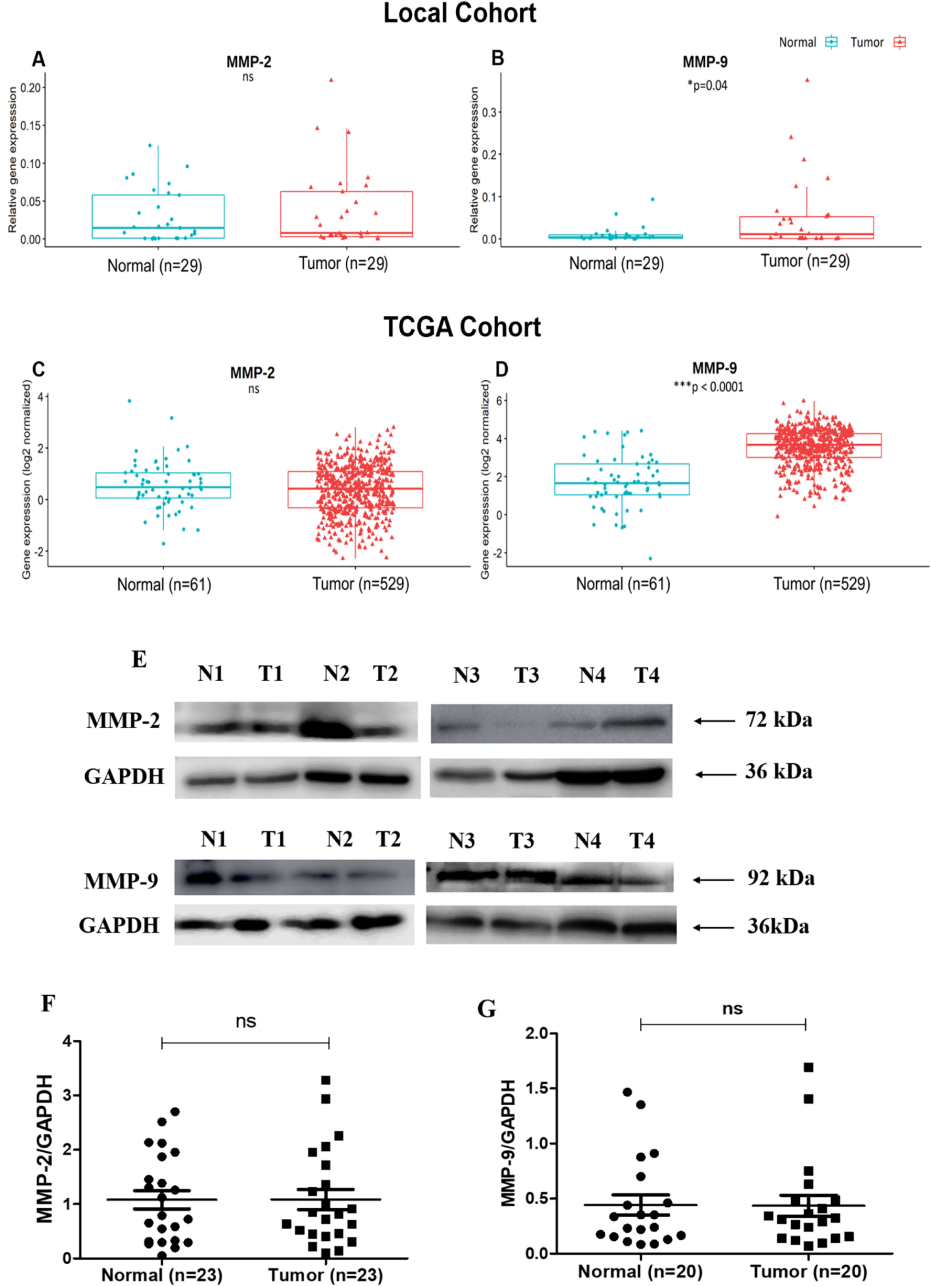
(**A**–**D**) Gene expression of metastasis markers in breast cancer patients (**A**) *MMP-2*, (**B**) *MMP-9* in local cohort (**C**) *MMP-2*, (**D**) *MMP-9* in TCGA cohort and (**E**–**G**) Protein expression of metastasis markers (**E**) Representative blots in adjacent normal (N) and tumor (T) tissues, (**F**) Densitometric analysis of MMP-2 and (**G**) MMP-9 levels in adjacent normal and tumor tissues.

Tumor samples showed significant upregulation in the levels of *MMP-9* (local cohort: (*p = 0.04); TCGA cohort: (***p < 0.0001) (Fig. 7B, D), while there was no difference in the levels of *MMP-2* between tumor and adjacent normal tissue in local as well as TCGA cohort (Fig. 7A, C)*.*

In the current studies, no significant difference in the protein expression of metastasis markers MMP-2 and MMP-9 was observed between tumor and adjacent normal tissue (Fig. 7E, F and G) (Supplementary Figs. 2 and 4).

##### Correlation of *CERK *and *SPHK1* with metastasis markers

**Figure 8:**
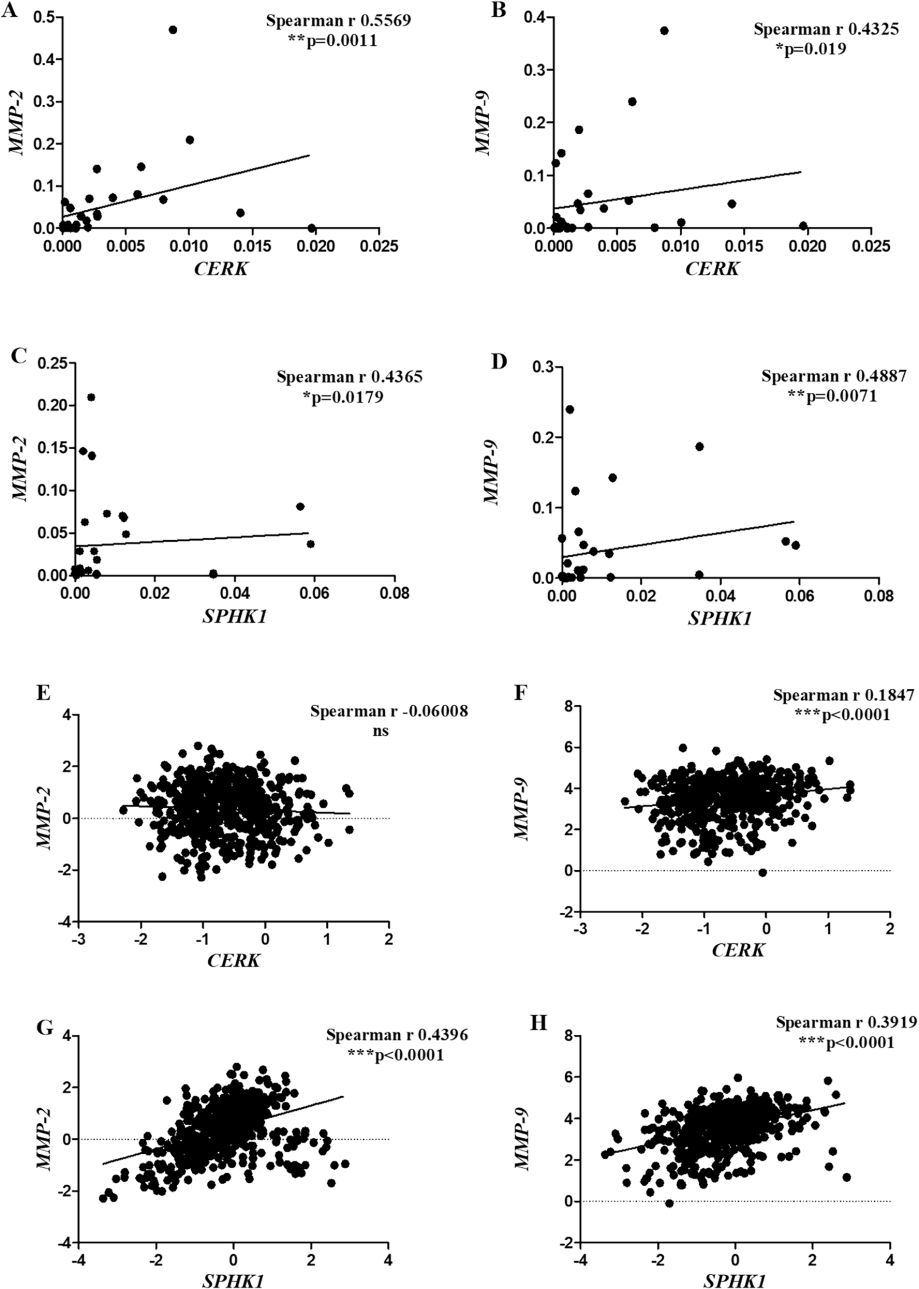
Correlation of *CERK* with (**A**) *MMP-2*, (**B**) *MMP-9* and *SPHK1* with (**C**) *MMP-2*, (**D**) *MMP-9* in local cohort and *CERK* with (**E**) *MMP-2*, (**F**) *MMP-9* and *SPHK1* with (**G**) *MMP-2*, (**H**) *MMP-9* in TCGA cohort.

In order to establish the role of sphingolipids in metastasis, their correlation with metastasis markers was determined. It was observed that *CERK* had a positive association with both the metastasis genes *MMP-2* (**p = 0.0011) (Fig. 8A) and *MMP-9* (*p = 0.019) (Fig. 8B). Expression of *SPHK1* was also found to be significantly correlated with *MMP-2* (*p = 0.0179) (Fig. 8C) and *MMP-9* (**p = 0.0071) (Fig. 8D). The correlation studies were further validated in the TCGA data cohort and similar results were found between the local and TCGA cohort. A significant positive association was observed between sphingolipid metabolism genes and metastasis markers (Fig. 8F, G and H) except for the *CERK* with *MMP-2* (Fig. 8E). These observations suggest that these sphingolipid metabolites might have a potential role in breast cancer metastasis.

##### Analysis of gene and protein expression of drug transporters

**Figure 9:**
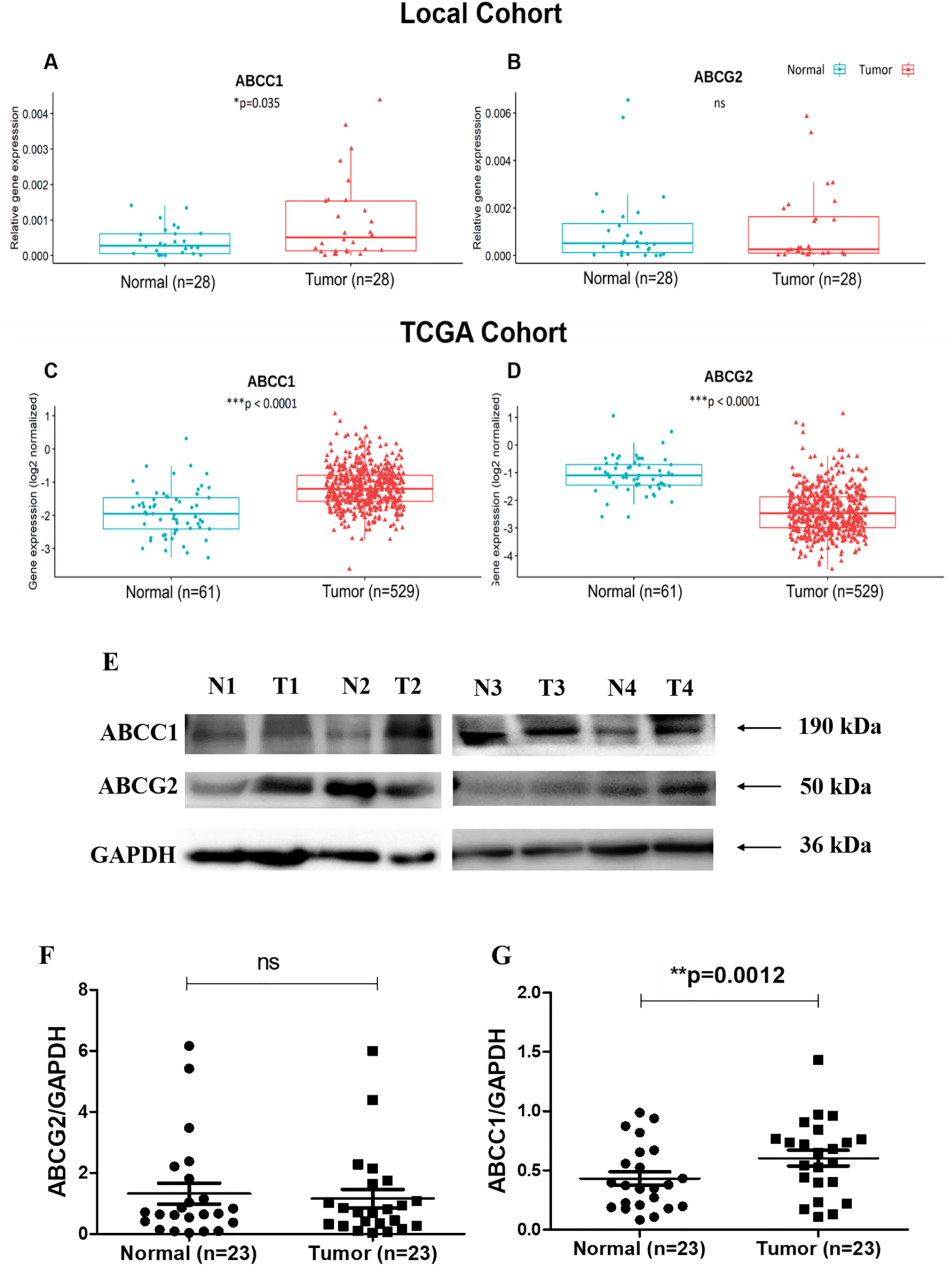
(**A**–**D**) Gene expression of drug transporters in breast cancer patients (**A**) *ABCC1*, (**B**) *ABCG2* in local cohort (**C**) *ABCC1*, (**D**) *ABCG2* in TCGA cohort and (**E**–**G**) Protein expression of drug transporters (**E**) Representative blots in adjacent normal (N) and tumor (T) tissues, (**F**) Densitometric analysis of ABCG2 and (**G**) ABCC1 levels in adjacent normal and tumor tissues.

Expression of two key drug transporters i.e., *ABCC1 and ABCG2* were analysed in tumor and adjacent normal breast tissues in local as well as TCGA cohort. The level of *ABCC1* was significantly upregulated in tumor tissue as compared to adjacent normal in local cohort (*p = 0.035) (Fig. 9A) and TCGA cohort (***p < 0.0001) (Fig. 9C). There was no statistically significant difference in the levels of *ABCG2* between tumor and adjacent normal tissue in the local cohort (Fig. 9B) whereas in TCGA cohort, it was found to be significantly higher in normal tissue as compared to tumor tissue (***p < 0.0001) (Fig. 9D).

Protein expression analysis revealed that there was no significant difference in the levels of ABCG2 between tumor and adjacent normal tissues (Fig. 9E, F). The levels of ABCC1 were found to be significantly higher in tumor tissue as compared to adjacent normal tissue (**p = 0.0012) (Fig. 9G) (Supplementary Figs. 3 and 4).

##### Correlation of *CERK *and *SPHK1* with drug resistance markers

**Figure 10:**
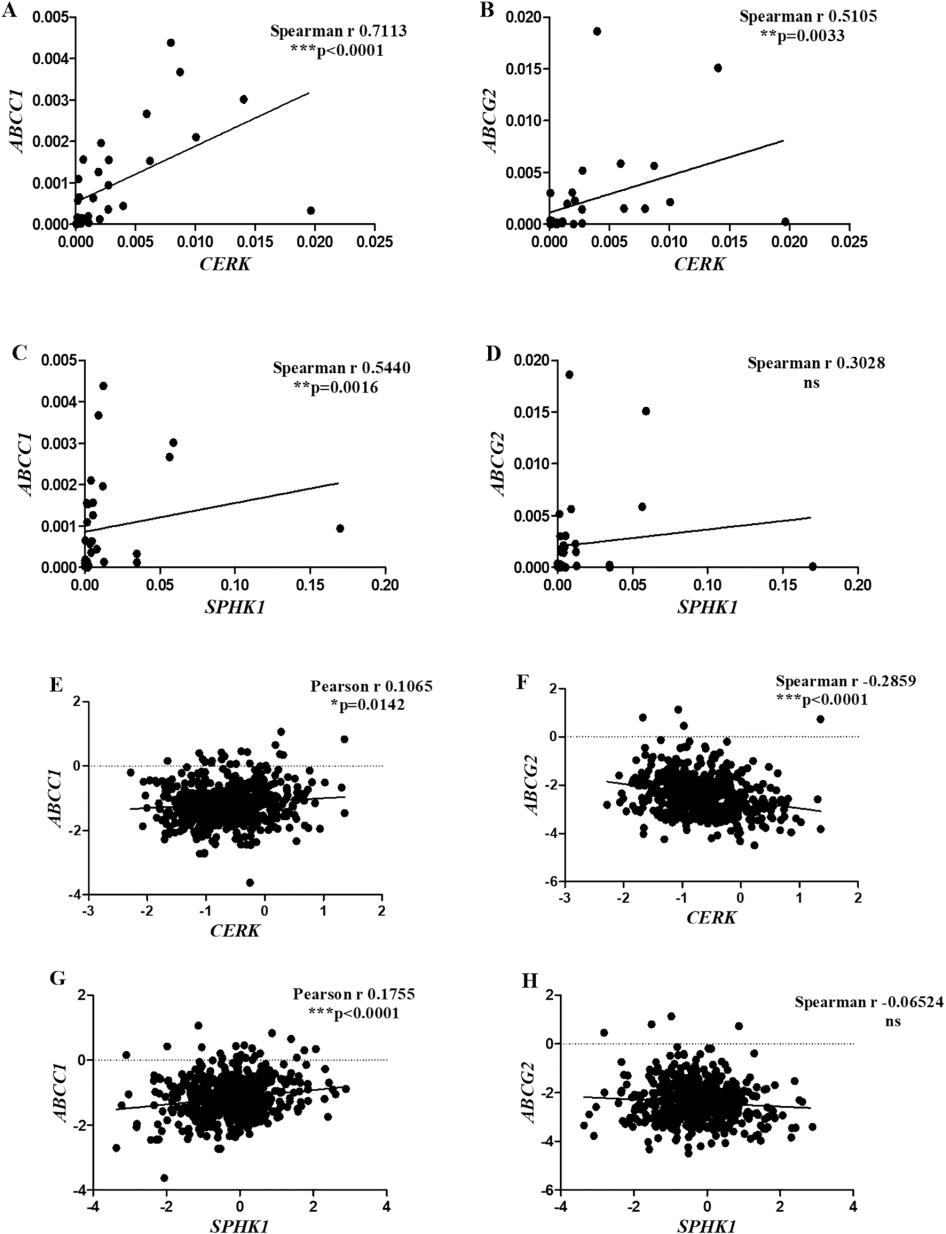
Correlation of *CERK* with (**A**) *ABCC1*, (**B**) *ABCG2* and *SPHK1* with (**C**) *ABCC1*, (**D**) *ABCG2* in local cohort and *CERK* with (**E**) *ABCC1*, (**F**) *ABCG2* and *SPHK1* with (**G**) *ABCC1*, (**H**) *ABCG2* in TCGA cohort.

The role of sphingolipids in drug resistance was examined by determining their correlation with drug resistance markers. It was revealed that *CERK* had a positive correlation with both the drug resistance genes *ABCC1* (***p < 0.0001) (Fig. 10A) and *ABCG2* (**p = 0.0033) (Fig. 10B)*.* Similarly, *SPHK1* was also found to be positively correlated to *ABCC1* (**p = 0.0016) (Fig. 10C). No association was observed between the levels of *SPHK1* and *ABCG2* (Fig. 10D). The local cohort studies were further validated in the TCGA data set. It was found that both *CERK* and *SPHK1* had a significant positive correlation with the drug transporter *ABCC1* (*p = 0.0142) (Fig. 10E) and (***p < 0.0001) (Fig. 10G), respectively. However, for *ABCG2*, a negative association with *CERK* (***p < 0.0001) (Fig. 10F) and no association with *SPHK1* was observed (Fig. 10H).

### Expression analysis of *SPNS2* and its correlation with *SPHK1*

**Figure 11:**
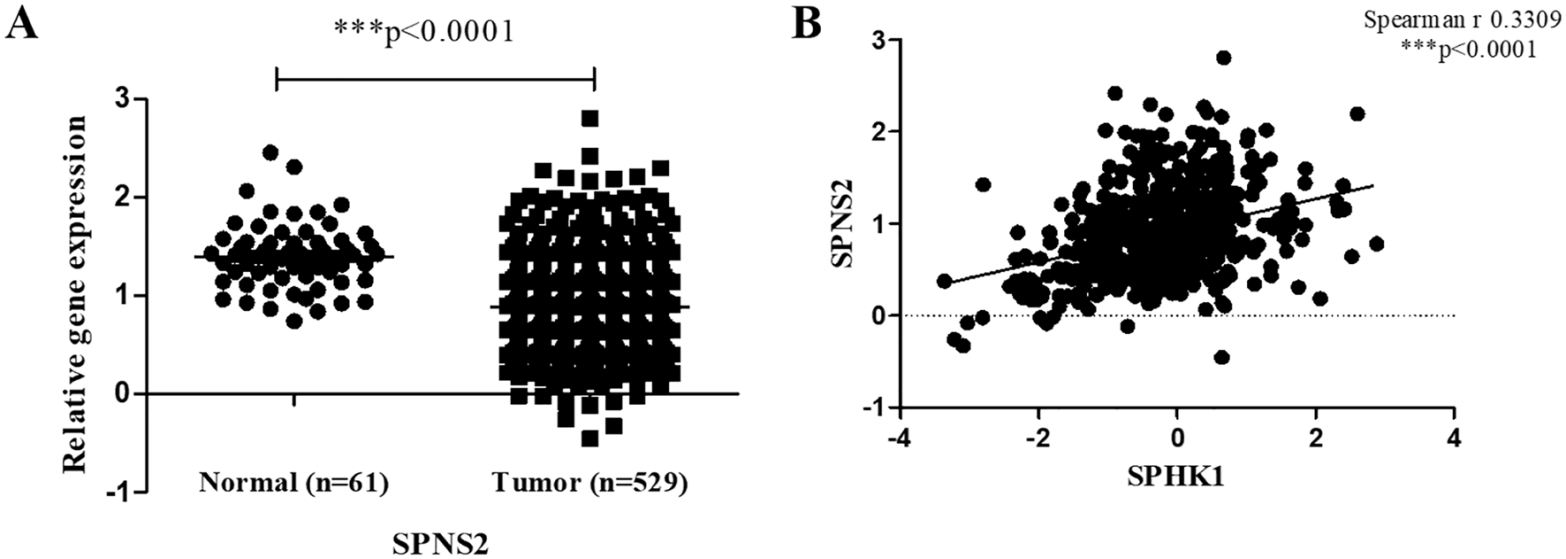
Expression of *SPNS2* in TCGA cohort. (**A**) Relative gene expression between adjacent normal and tumor tissue, (**B**) Correlation with *SPHK1*.

The S1P transporter *SPNS2* was also observed in the interaction network analysis (Supplementary Table S1). Since, *SPNS2* was a transitive protein and not the primary interactor of target genes, its expression was analysed only in the TCGA cohort and was correlated with *SPHK1*. It was observed that the *SPNS2* levels were downregulated in tumor tissue as compared to adjacent normal tissue (***p < 0.0001) (Fig. 11A). Correlation analysis revealed that the level of *SPNS2* were positively associated with *SPHK1* levels in breast tumor tissues (***p < 0.0001) (Fig. 11B).

## Discussion

Sphingolipids represent the largest class of bioactive lipids associated with various aspects of tumorigenesis such as cell growth, proliferation, migration and drug resistance^19,20^. More recently, the clinical significance of sphingolipid-related biomarkers has acquired a forefront in cancer diagnosis and prognosis. In a previously published report, we found an elevation in the levels of sphingolipid metabolites CerP, S1P and SM in tumor tissues of breast cancer patients. There was also an upregulation in the expression of *CERK* and *SPHK1* genes, involved in the synthesis of CerP and S1P metabolites. However, limited information is available on association of *CERK* and *SPHK1* with clinical outcome in patient samples. In the present study, we aimed to determine association of these genes with clinicopathological characteristics as well as other cancer cellular processes in order to determine their clinical relevance in breast cancer.

*CERK* is a lipid kinase that regulates cellular processes such as proliferation, migration and inflammation. High expression of *CERK* has been reported to contribute to cell migration and invasion in metastatic breast cancer cells^21^. An earlier report has also highlighted the significance of *CERK* signaling in cancer migration and proliferation in human pancreatic cancer cells^22^. The inhibition of *CERK* by NVP-231 was found to decrease the rate of proliferation in breast and lung cancer cells^23^. *CERK* has also been reported earlier to promote tumor cell growth, survival and mammary tumor recurrence^24,25^. However, there is no study defining the significance of *CERK* among various clinical subgroups in breast cancer. The present study determined the association of *CERK* to various clinicopathological variables. We observed a significant upregulation in the expression of *CERK* in ER positive, nodal positive and late-stage breast cancer patients. A significant positive correlation was also observed between *CERK* levels and Ki67 index of breast cancer patients. High expression of *CERK* was also associated with poor OS in nodal positive and late-stage breast cancer patients. A previous study has also linked the increased expression of *CERK* with poor recurrence free survival in ER-negative breast cancer patients^26^. We further validated the gene expression results at post-transcriptional level. Consistent with the previous findings, increased protein expression of CERK was observed in tumor tissues as compared to adjacent normal tissues. Above studies therefore, reinforce the significance of CERK in discriminating the tumor tissue from adjacent normal tissues in breast cancer patients. High expression of *CERK* in lymph node positive and late-stage cancer patients and its association to poor OS further strengthen its potential as a prognostic biomarker in breast cancer patients.

The clinical correlations of *SPHK1* were also assessed in the current study. Similar to the *CERK*, the levels of *SPHK1* were found to be significantly high in patients with nodal metastasis and advanced stage of breast cancer. Our findings are in agreement with a previous study that reported an association between high *SPHK1* expression and tumor size, lymph node metastasis and tumor-node-metastasis in papillary thyroid carcinoma^27^. The level of lymph node metastasis has been recognized as a major determinant of staging and prognosis in breast cancer^28,29^. Increased expression of *SPHK1* was also found to corelate with clinical stage and distant metastasis of nasopharyngeal carcinoma^30^. In yet another finding, primary sites of colon cancers with metastases exhibited progressively high levels of *SPHK1* than in those without metastases^31^. Inhibiting *SPKH1* significantly decreased the serum S1P levels which in turn reduced tumor burden of the primary tumor, lymph node and lung metastases in murine model of metastatic breast cancer^32^.

Overexpression of *SPHK1* has also been reported to significantly enhance the proliferation and invasion of papillary thyroid carcinoma cell lines^27^. An earlier study has also documented that increased *SPHK1* expression is associated with increased Ki-67 in human nasopharyngeal carcinoma specimens^33^. In line with the previous studies, we also observed a positive significant association between *SPHK1* expression and the proliferation marker Ki67. Similarly, a recent finding suggests that oral squamous cell carcinoma patients with high *SPHK1* expression exhibit a higher Ki-67 labeling index than the patients with low *SPHK1*. However, the authors failed to establish the statistical association between *SPHK1* and Ki-67 index^34^. In addition to their role in proliferation and invasion, increased expression of *SPHK1* also contributes to poor OS of triple-negative breast cancer patients^35^. Other studies have also demonstrated an association between high *SPHK1* levels and poor prognosis in patients with glioblastoma^36^ or breast cancer^37^. The elevated levels of *SPHK1* were also found to correlate with shorter OS in hepatocellular carcinoma^38^. In the present study, the expression of *SPHK1* did not show any impact on the overall prognosis of the disease. Consistent with the gene expression analysis, the present study also reports a significant elevation in the levels of SPHK1 proteins in tumor tissues as compared to adjacent normal. Similar to our findings, SPHK1 protein expression was found to be significantly upregulated in hepatocellular carcinoma cell lines^38^. The above observations clearly indicate that *SPHK1* exhibit a good diagnostic ability in differentiating the lymph node metastasis and TNM stages and might serve as a potential biomarker to predict the clinical outcome in breast cancer patients.

The results of the current studies however, could not be validated in TCGA cohort wherein, no correlation was observed between the expression of *CERK*, *SPHK1* and various clinical subgroups. The lack in significance could be attributed to racial/ethnical disparity of the TCGA patient cohort. A recent study also found that for the majority of cancers, there was a difference in the clinical characteristics between TCGA and general population cancer^39^.

To establish the significance of sphingolipids in drug resistance and metastasis of breast cancer patients, a regulatory network of *SPHK1* and *CERK* with metastasis and drug resistance markers was inferred and analyzed. *CERK* and *SPHK1* had smaller shortest paths across each other as well as across MMP-2, MMP-9 and ABCC1, ABCG2. Importantly, *CERK* was not highly connected nor was highly between in the network, but it was functionally close with other candidates, indicating it to be playing major role in maintaining the regulatory interactions during breast cancer. Overall, network analysis showed that all of the six candidate genes were closely correlated and might regulate each other during cancer processes. The results of the above observations were further validated in local as well as TCGA cohort.

### Association of *CERK* and *SPHK1* with metastasis markers

In the present study, we measured the expression of *MMP-2* and *MMP-9* in breast cancer patients and determined their correlations with *SPHK1* and *CERK* in local as well as TCGA cohort. Expression of *MMP-2* was not found to be differentially regulated between the tumor and adjacent normal tissue in both the cohort. These observations are in alliance with a recent finding that showed no significant difference in *MMP-2* levels between breast cancer and normal tissues^40^. On the contrary, the levels of *MMP-2* were significantly upregulated in colorectal cancer tissues than in normal tissues^41^.

The current study reports significantly high levels of *MMP-9* in tumor tissues as compared to adjacent normal tissues in local as well as TCGA cohort. Similarly, a significant elevation in *MMP-9* expression has also been reported in tumoral tissues than adjacent non-tumoral tissues in breast cancer^40^ and papillary thyroid cancer patients^42^. However, there was no difference in the protein expression of MMP-2 and MMP-9 between tumor and adjacent normal tissues. The lack in significance may be attributed to small sample size of the study.

To establish the role of sphingolipids in breast cancer metastasis, we analyzed the associations of *MMP-2* and *MMP-9* with *SPHK1* and *CERK* expression. In local cohort, there was a significant positive correlation of *SPHK1* and *CERK* levels with *MMP-2* and *MMP-9*. Similar set of results were also observed in TCGA cohort except for the correlation of *CERK* with *MMP-2*. Evidence from past literature demonstrates that *SPHK1* enhances the production of *MMP-2* and *MMP-9* and thereby promotes tumor proliferation and invasion of colon cancer^9^. High expression of *SPHK1* was also shown to increase *MMP-2/MMP-9* mRNA levels in head and neck squamous cell carcinoma^43^. However, to our knowledge, no previous report has determined the association of C*ERK* with *MMP-2* and *MMP-9*. These findings clearly suggest that *CERK* and *SPHK1* might contribute to the breast cancer metastasis by modulating the expression of *MMP-2* and *MMP-9*.

### Association of *CERK* and *SPHK1* with drug resistance markers

A plethora of mechanisms are reported to be involved in the process of MDR of which the best described is overexpression of members of the ABC transporter superfamily. Evidence from previous research stipulate ABCB1, ABCC1 and ABCG2 as the most likely targets in the MDR development^5,44^. To confirm the above hypothesis, we analyzed the expression of two drug transporters i.e., ABCC1 and ABCG2 in breast cancer patients and determined their association with *SPHK1* and *CERK* in local as well as TCGA cohort. A significant upregulation of *ABCC1* was observed in tumor tissues in both local as well as TCGA cohort. The protein levels of ABCC1 were also found to be high in tumor tissues as compared to adjacent normal tissues. Similar to our findings, high levels of *ABCC1* in lymph node positive patients were suggestive of their potential role in breast cancer metastasis^45^. Another recent study in breast cancer found that expression level of *ABCC1* in tumor tissue of patients responding to chemotherapy was significantly lower compared to non-responding patients^46^. Elevated levels of *ABCC1* have been reported to be associated with clinical drug resistance in several malignancies such as lung, esophageal, leukemia and childhood neuroblastoma^47–50^. These observations suggest that *ABCC1* can be envisioned as likely determinant of treatment response to various chemotherapeutic drugs in breast cancer patients.

In the current study, the levels of *ABCG2* were downregulated in tumor tissues in local as well as TCGA cohort. However, the difference was not statistically significant in local cohort studies. Further, there was no difference in the protein expression between tumor and adjacent normal breast tissues. Albeit, small sample size of the local cohort is the main limitation associated with this study. Further investigations with large cohorts will be needed to illustrate the clinical utility of ABCG2 in breast cancer patient population.

Correlation studies revealed that *ABCC1* levels were positively associated with *CERK* and *SPHK1* expression in both the cohorts. A recent study has also shown that increased export of S1P via *ABCC1* enhances the transcription of *SPHK1* resulting in more S1P formation in human MCF-7 breast cancer cells^51^. For *ABCG2*, a positive correlation with *CERK* in local cohort and a negative correlation in TCGA cohort was observed. However, no significant correlation was observed between *ABCG2* and *SPHK1* in both the cohorts.

Interaction network analysis also led to the identification of S1P transporter SPNS2. The levels of *SPNS2* were found to be downregulated in tumor tissue as compared to adjacent normal tissue in TCGA cohort. Similar to our findings, a recent study suggests that *SPNS2* expression was markedly lower in ten kinds of tumor (including breast invasive carcinoma) as compared to adjacent non-cancerous samples. The authors reported that the levels of *SPNS2* were downregulated in colorectal cancer and were associated with poor differentiation, advanced pTNM stage and poor prognosis of the disease^52^. Being a S1P transporter, the levels of *SPNS2* were found to be positively associated with *SPHK1* expression in tumor tissue in the current study. However, high expression of *SPHK1* and low expression of *SPNS2* in tumor tissue suggest that more S1P is being made but not transported from the cell via *SPNS2*. Since the current study reports the role of S1P in tumor proliferation, we hypothesize that S1P is being transported from the cell via other transporters. The possible mechanism for S1P export might be through ABC transporter *ABCC1* as it is also upregulated in the current study. Similar observations were reported in an earlier study, where export of S1P via ABCC1 was found to amplify the S1P axis involved in breast cancer progression and metastasis^51^. However, future in vitro studies are required to explore the role of *SPNS2* and its association to S1P axis in breast cancer patients.

Numerous studies have elucidated dysregulations in the levels of sphingolipid metabolites in various cancer types. However, the clinical relevance of sphingolipids as biomarkers in breast cancer patients remains elusive. The present study underscores the significance of sphingolipids in breast cancer patients in terms of diagnosis, prognosis and clinical correlations. High levels of *SPHK1* and *CERK* in nodal positive and late-stage cancer patients as well as association of high *CERK* expression to poor patient survival are indicative of their potential as diagnostic and prognostic biomarkers. In addition, positive correlation of sphingolipid genes with Ki67 index, metastasis and drug resistance markers reinforces the assumption that these sphingolipids might have a predictive role in breast cancer proliferation, migration and drug resistance.

We acknowledge that small sample size of the local cohort is the main limitation of the present study. However, we have tried to validate gene expression analysis in large TCGA cohort. We were also unable to correlate the gene or protein expression with the patient survival in local cohort due to lack of follow up data. Further investigation in another independent large cohort is essential to confirm the present findings. Additionally, mechanistic evidences also need to be uncovered in order to exploit these findings for development of therapeutics.

## Materials and methods

### Chemicals and solutions

PureLink RNA isolation kit, RevertAid First Strand cDNA Synthesis Kit, DNase I enzyme, PowerUp SYBR Green Master Mix were purchased from Thermo Fisher Scientific (Waltham, Massachusetts, United States). RNA*later*, TRIzol Reagent and Bradford reagent were purchased from Sigma-Aldrich (St. Louis, Missouri, United States). Primers designed for the genes were purchased from Integrated DNA Technologies (Coralville, Iowa, United States). Primary antibodies against SPHK1 (NBP2-20472); CERK (NB100-2911); ABCC1 (NB400-156); ABCG2 (NBP2-22124); MMP-2 (NBP2-27208SS); MMP-9 (NBP2-13173SS) were purchased from Novus Biologicals (Centennial, Colorado, United States). Monoclonal primary antibody against glyceraldehyde 3-phosphate dehydrogenase (GAPDH) (AM4300) was received from Thermo Fisher Scientific (Waltham, Massachusetts, United States). HRP-linked anti rabbit secondary antibody (7074) was purchased from Cell Signaling Technology (Danvers, Massachusetts, United States) and HRP- linked anti-mouse (SC-2005) was obtained from Santa Cruz Biotechnology (Dallas, Texas, United States). Clarity ECL Western Blotting Substrate was purchased from Bio-Rad (Hercules, CA, USA). All other chemicals used were of molecular biology grade.

### Study design and ethics statement

The present study comprised of two different cohorts: local and TCGA. Both the cohorts involved the analysis of tumor and adjacent normal breast tissues. The study was performed in accordance with 1975 Declaration of Helsinki. Informed consent was obtained from the patients for the collection of specimens and clinical data. Ethical approval for the study was obtained from Institutional Ethics Committee, PGIMER (IEC-12/2017-787) and Panjab University Institute Ethics Committee (PUIEC/2019/154/A/01/03).

### Sample collection

The tumor and adjacent normal tissues were obtained from the breast cancer patients who underwent surgical resection at Department of General Surgery, PGIMER, Chandigarh. The current study is an extension of our previous study wherein a total of 31 patients constituted the local cohort^14^. Clinical details were recorded and a pathological staging was done for all patients. Patient clinical characteristics included age, tumor type, pathological grade, pathologic T, N, M stage and Ki67 index. The TNM staging was done by the experienced pathologists according to the American Joint Committee on Cancer (AJCC) 8th edition. Breast tissues were also classified into estrogen receptor (ER), progesterone receptor (PR) and human epidermal growth factor receptor 2 (Her2) subtypes based on immunohistochemical (IHC) characteristics. The tissue samples were stored at − 20 °C in RNA later for RNA isolation and − 80 °C for protein isolation.

### Histopathological analysis

The breast tissues were dissected, flushed with ice cold phosphate-buffered saline (PBS) and fixed in 10% neutral buffered formalin. Tissue was paraffin embedded and cut into sections of 5 µm thickness using a microtome. The sections were transferred to glass slides and stained with standard H&E - staining method. The slides were photomicrographed (3 random fields from each slide) using Nikon Eclipse 80i microscope (Nikon, Kawasaki, Japan).

### RNA extraction and cDNA synthesis

Total RNA was extracted from the tissue samples using PureLink RNA Mini Kit (Thermo Fischer Scientific Waltham, Massachusetts, United States) according to the manufacturer’s instructions. The kit employs an organic extraction followed by immobilization of RNA on glass-fibre filters to purify total RNA. The purified RNA was stored at − 20 °C till further use. The integrity of RNA was checked on 2% agarose using gel electrophoresis. The concentration and purity of RNA was checked using NanoDrop 2000c Spectrophotometer. A total of 1 μg RNA was taken for cDNA synthesis. Prior to this, a treatment with DNase I enzyme was given to remove any possible genomic contamination. cDNA synthesis was carried out using Revert Aid First Strand cDNA synthesis kit purchased from Thermo Fischer Scientific (Waltham, Massachusetts, United States). The quality of the synthesized cDNA was checked by amplification of the housekeeping gene *GAPDH*.

### Interaction network inference and analysis

A PPI network comprising *CERK* and *SPHK1*, metastasis markers and drug resistance markers was inferred by integrating physical PPIs and gene co-expression regulations among candidates as well as their corresponding interacting partners; physical interactions were obtained from the STRING database (string-db.org) while gene co-expression regulations were identified by processing gene expression data (BRCA-TCGA) obtained from the Broad Institute (gdac.broadinstitute.org). Topological analyses of the network were performed to assess the centrality of candidates based on their highest connectivity/degree (k), which represents network ‘hubs’, and BC which represents the network ‘bottlenecks’^53^. The biological relevance of centrality measures in prioritizing candidates was also evaluated by comparing the distribution of essential and non-essential genes in the interaction network. The shortest paths across *CERK* and *SPHK1* and metastasis and drug resistance markers in the network were computed using the Dijkstra’s algorithm. All network analyses and visualizations were performed in R-igraph (v 1.2.6; igraph.org/r) and Cytoscape (v 3.8.2; cytoscape.org) software.

### Primer designing for target genes

Primer designing for the genes of interest and the housekeeping gene was carried out using Primer-blast software. The primers were further assessed by Beacon Designer Free edition for the presence of secondary structures. The sequence of primers used is given in the Table 1.

**Table 1:** List of primers designed and their sequences*.*

_Gene_	_Forward primer_	_Reverse primer_	_Amplicon size_	_Annealing temperature_
_*SPHK1*_	_5′-AGGCTGAAATCTCCTTCACGC-3′_	_5′-GTCTCCAGACATGACCACCAG-3′_	_113 bp_	52 °C
_*CERK*_	_5′-TGGTTGGGTCTTGCCAGATAC-3′_	_5′-ACTTCCCACAGACGACTTGC-3′_	_250 bp_	52 °C
_*ABCC1*_	_5’-AGCCGGTGAAGGTTGTGTAC -3’_	_5’-TGACGAAGCAGATGTGGAAG -3'_	_360 bp_	52 °C
_*ABCG2*_	_5’- TATAGCTCAGATCATTGTCACAGTC-3’_	_5’-GTTGGTCGTCAGGAAGAAGAG-3’_	_124 bp_	52 °C
_*MMP-2*_	_5'-CTCATCGCAGATGCCTGGAA-3'_	_5'-TTCAGGTAATAGGCACCCTTGAAGA-3'_	_104 bp_	53 °C
_*MMP-9*_	_5'-ACGCACGACGTCTTCCAGTA-3'_	_5'-CCACCTGGTTCAACTCACTCC-3'_	_94 bp_	53 °C
_*GAPDH*_	_5′-ACCCACTCCTCCACCTTTGA-3′_	_5′-CTGTTGCTGTAGCCAAATTCGT-3′_	_101 bp_	60 °C

### Quantitative polymerase chain reaction (qPCR)

qPCR analysis was carried out to determine the mRNA expression of target genes in tumor and adjacent normal breast tissues. cDNA was diluted to a final concentration of 10 ng/μl. The annealing temperature for all the genes was standardized using gradient PCR. The reactions were performed in duplicates in Applied Biosystems Quant Studio 3 Real Time PCR System (Waltham, MA, USA) using Powerup SYBR green mastermix (Thermo Fisher Scientific, Waltham, Massachusetts, United States). The average Ct values for each sample were recorded and normalized with GAPDH as endogenous control. The relative gene expression was calculated using 2^−ΔΔCt^ method.

### Protein expression analysis

The expression of target proteins was studied using the standard western blotting method given by Towbin et al.^54^. The extraction of total proteins from the tumor and adjacent normal breast tissues was carried out in ice cold RIPA buffer containing 0.2% EZBlock Universal Protease and Phosphatase Inhibitor Cocktail (BioVision, Inc. Milpitas, CA, United States). The protein concentration of total cell lysates was determined by standard Bradford method^55^. A total of 50 μg of protein was separated on 10% SDS–polyacrylamide gel and electro-transferred to polyvinylidenedifluoride membranes (Bio-Rad, Hercules, United States). After blocking with 3% BSA, the membranes were incubated with primary antibodies for SPHK1 (1:1000), CERK (1:1000), MMP-2 (1:1000), MMP-9 (1:1000), ABCC1 (1:500), ABCG2 (1:1000) and GAPDH (1:2500) overnight with shaking at 4 °C. The membranes were then incubated with horseradish peroxidase (HRP) labeled goat anti-mouse (1:3000) and antirabbit (1:5000) secondary antibodies at room temperature for 1 h. The immunoblots were visualized on Image Quant LAS 500 (GE Healthcare, Chicago, Illinois, United States) using Enhanced Chemiluminescence Detection Kit. Densitometry analysis was performed using the AlphaView Software. Since the blots were cut prior to hybridization with antibodies, it was difficult to provide full length membrane images.

### TCGA gene expression

Gene expression data pertaining to breast invasive carcinoma (TCGA abbreviation BRCA*)* was retrieved from the Firehose portal of Broad Institute (gdac.broadinstitute.org); this dataset comprises lowest-normalized level-3 microarray data (Agilent 244 K [G4502A]) from 529 tumor and 61 adjacent normal tissues. All statistical analysis and image rendering were performed in R 3.4.4 statistical environment (www.r-project.org).

### Statistical analysis

Statistical analysis was performed using Graphpad Prism 5 software (La Jolla, CA, United States), SPSS statistics 21(IBM, Armonk, NY, United States) and MedCalc for Windows, version 15.0 (Ostend, Belgium). The continuous data were represented as Mean ± SEM. To detect and exclude any potential outliers, we considered the following points (1) either the expression values in tumor and normal are greater than value 75th + 1.5 × (value75th − value 25th) or (2) lesser than value 25th − 1.5 × (value 75th − value 25th).

For intergroup comparisons, Wilcoxon t-test (non-parametric) or paired t-test (parametric) was used to calculate the difference in expression between tumor and adjacent normal tissues. To compare sub-groups within same group and to calculate difference between the expression of tumor and normal samples in TCGA datasets Mann–Whitney *U* test (non-parametric) or unpaired t-test (parametric) was used. Correlations were carried out using Pearson's (parametric) or Spearman's (non-parametric) coefficient. Kaplan–Meier survival curves and log-rank test were used for survival analysis in TCGA data. All *p* values were two-sided and were considered significant when < 0.05.

## Supplementary Information

Supplementary Information.

## Data Availability

The datasets used and/or analysed during the current study available from the corresponding author on reasonable request.
