# Supplementary material for: Clinical relevance of CERK and SPHK1 in breast cancer and their association with metastasis and drug resistance
Source: Sci Rep. 2022 Oct 29;12:18239. doi: 10.1038/s41598-022-20976-0 (PMC9617946; doi:10.1038/s41598-022-20976-0)
Supplement: Supplementary file 1 — Supplementary Information. [file 41598_2022_20976_MOESM1_ESM.docx]

**Clinical relevance of CERK and SPHK1 in breast cancer and their association with metastasis and drug resistance**

**Priyanka Bhadwal^1^, Vinay Randhawa^1,2^, Kim Vaiphei^3^, Divya Dahiya^4^* and Navneet Agnihotri^1^***

1. Department of Biochemistry, Panjab University, Chandigarh, India
2. Cardiovascular Division, Department of Medicine, Brigham and Women's Hospital, Harvard Medical School, Boston, MA, USA
3. Department of Histopathology, PGIMER, Chandigarh, India
4. Department of General Surgery, PGIMER, Chandigarh, India

*Corresponding Authors E-mail: [agnihotri.navneet@gmail.com](mailto:agnihotri.navneet@gmail.com), [dahiyadivya@yahoo.com](mailto:dahiyadivya@yahoo.com)

Contact Nos- 9815992186, 9501583520

**R1. Biological relevance of centrality measures**

The biological relevance of these measures in prioritizing candidates was also evaluated by comparing the distribution of essential and non-essential genes in the interaction network using three-step approach: (i) classification of network genes (n=506) into essential (n=223 [44.07%]) and non-essential (n=283 [55.93%]) subsets, based of 6,936 essential human genes compiled from OGEE (Online GEne Essentiality database; https://v3.ogee.info), (ii) computing the percentages of essential and non-essential genes in top- and bottom-ranked genes, based on centrality measures.

Briefly, genes in the network were classified into essential (n=223 [44.07%]) and non-essential (n=283 [55.93%]) sets based of 6,936 essential human genes compiled from OGEE, a gene essentiality database that includes large-scale experimentally tested essential genes for various species. Next, the percentages of essential genes were computed in top- and bottom-ranked genes, for which the definition of centrality was varied between top-5 and top-100 (step size=5). It was identified that the top-k and BC genes were characterized by significantly higher percentage (Top-k_Essential_=71.97; Top-k_Non-essential_=28.03; Top-BC_Essential_=65.00; Top-BC_Non-essential_=35.00) of essential genes contrary to bottom-ranked genes having low percentage (Bottom-k_Essential_=27.10; Bottom-k_Non-essential_=72.90; Bottom-BC_Essential_=25.47; Bottom-BC_Non-essential_=74.53) of essential genes. Overall, these findings indicated essential genes tend to be topologically highly central as compared to others.

**Table S1**. The number of shortest paths and transitive proteins among candidates

| **Protein1** | **Protein2** | **No. of Shortest Paths** | **Transitive proteins** |
| --- | --- | --- | --- |
| *CERK* | *SPHK1* | 2 | *ACER1* |
| *CERK* | *MMP-2* | 3 | *ACER2, ITGB1* |
| *CERK* | *MMP-9* | 2 | *ASAH1* |
| *CERK* | *ABCC1* | 2 | *S1PR2* |
| *CERK* | *ABCG2* | 2 | *SPNS2* |
| *SPHK1* | *MMP-2* | 2 | *AKT1* |
| *SPHK1* | *MMP-9* | 2 | *AKT1* |
| *SPHK1* | *ABCC1* | 1 | - |
| *SPHK1* | *ABCG2* | 2 | *ABCC1* |


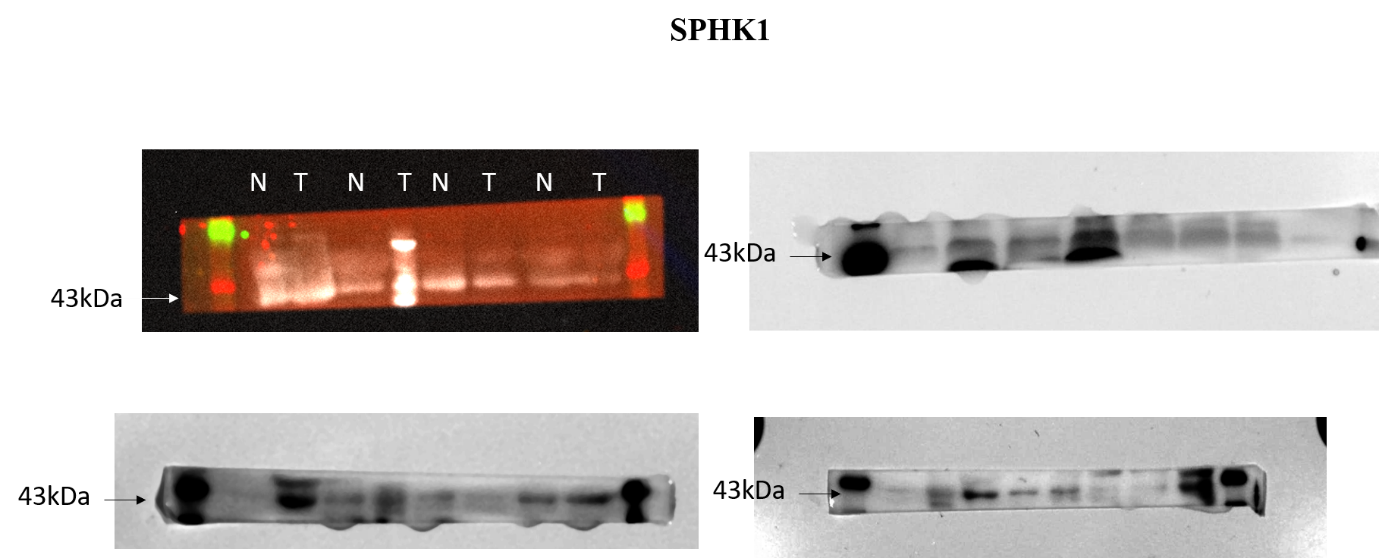


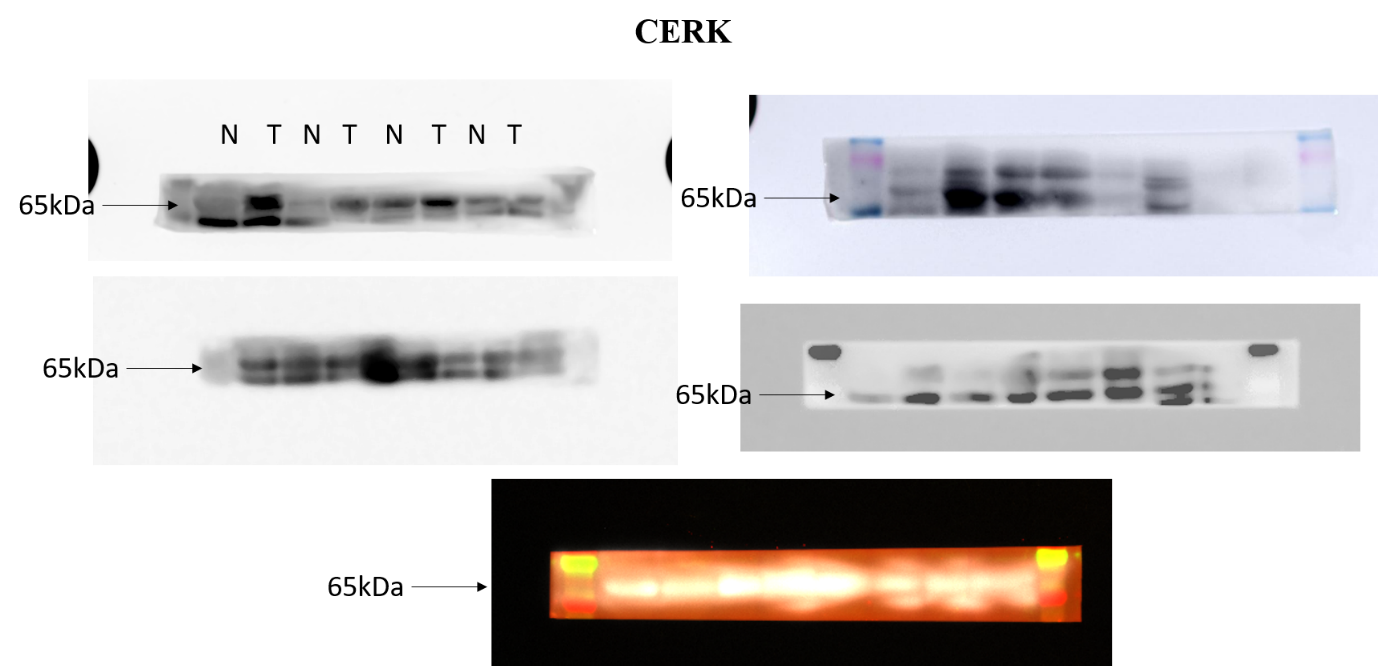


**Supplementary Figure 1.** Uncropped blot images showing SPHK1 and CERK


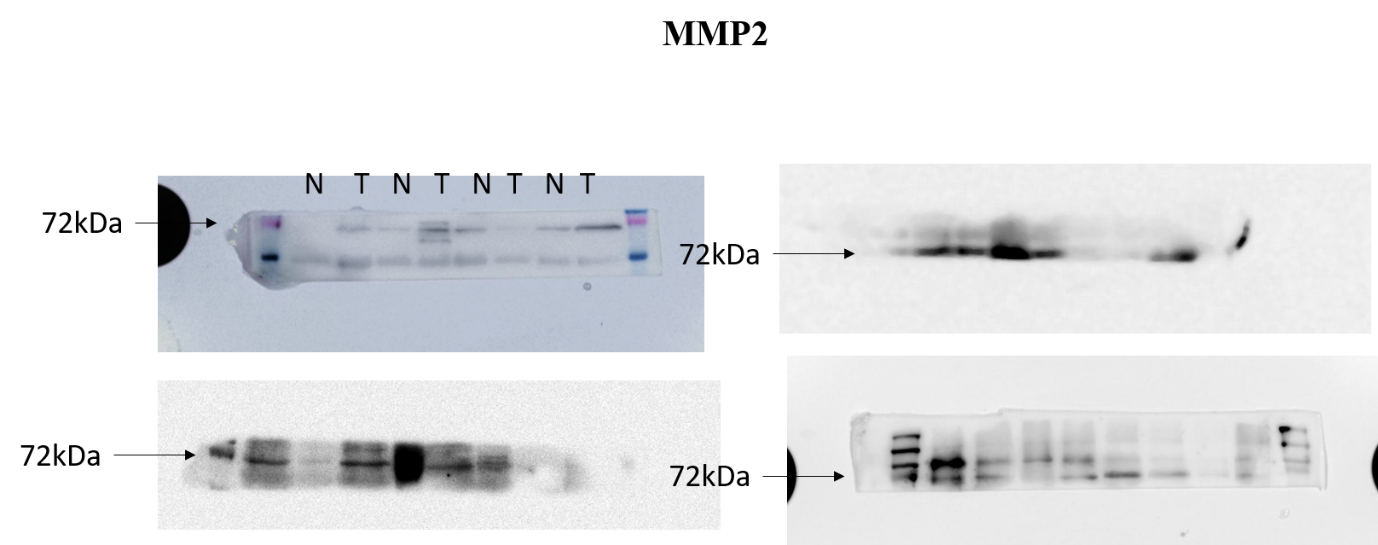


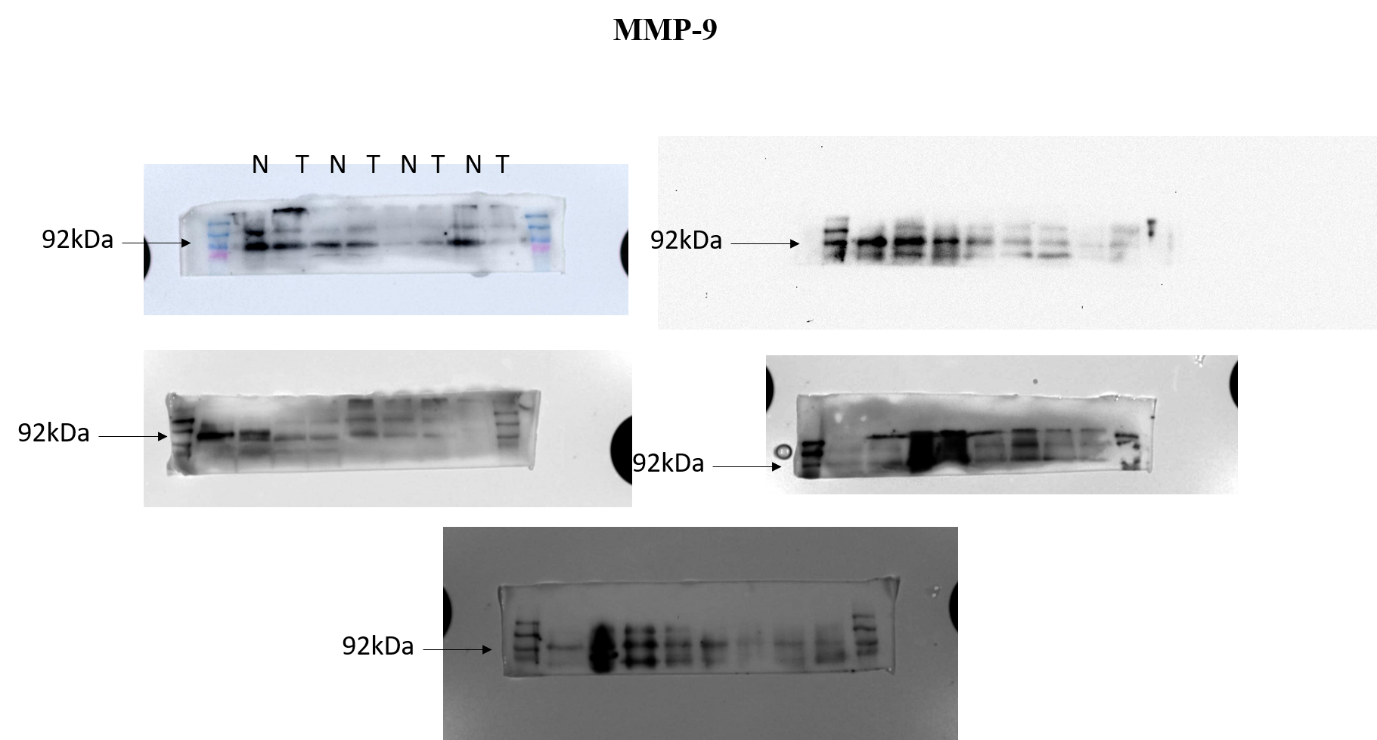


**Supplementary Figure 2.** Uncropped blot images showing MMP-2 and MMP-9


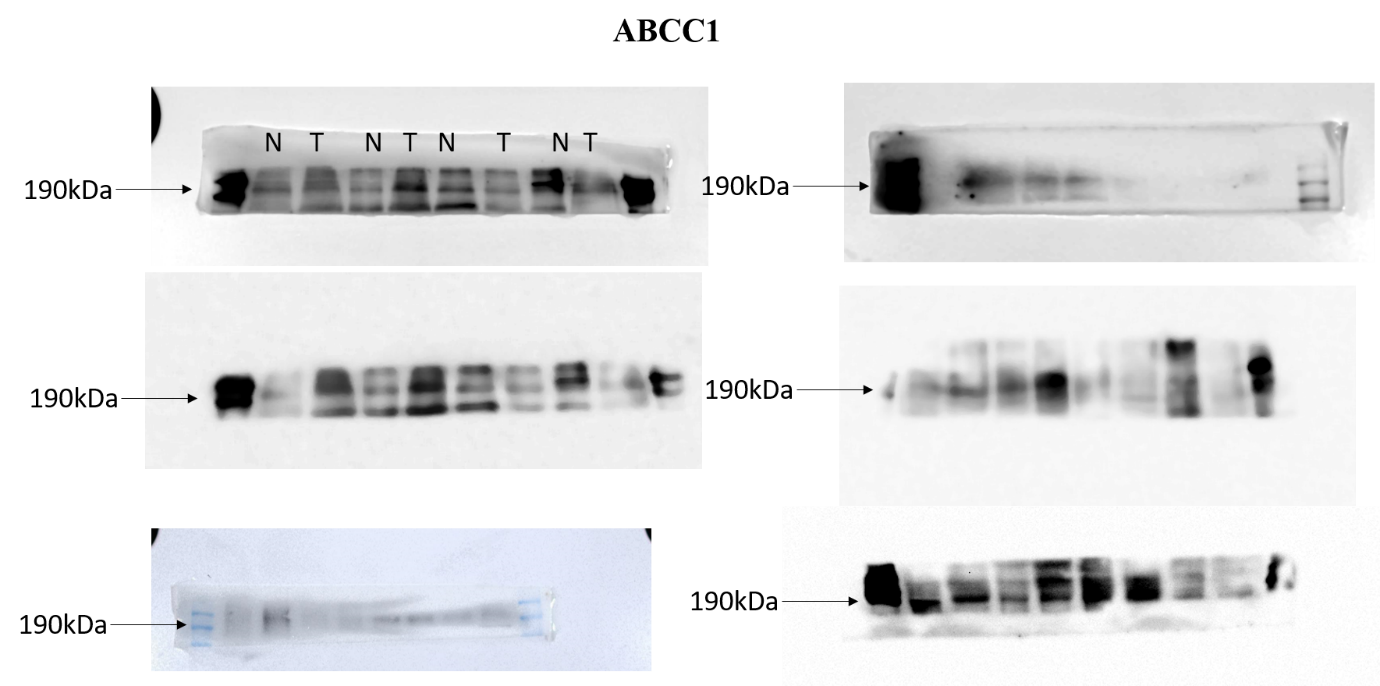


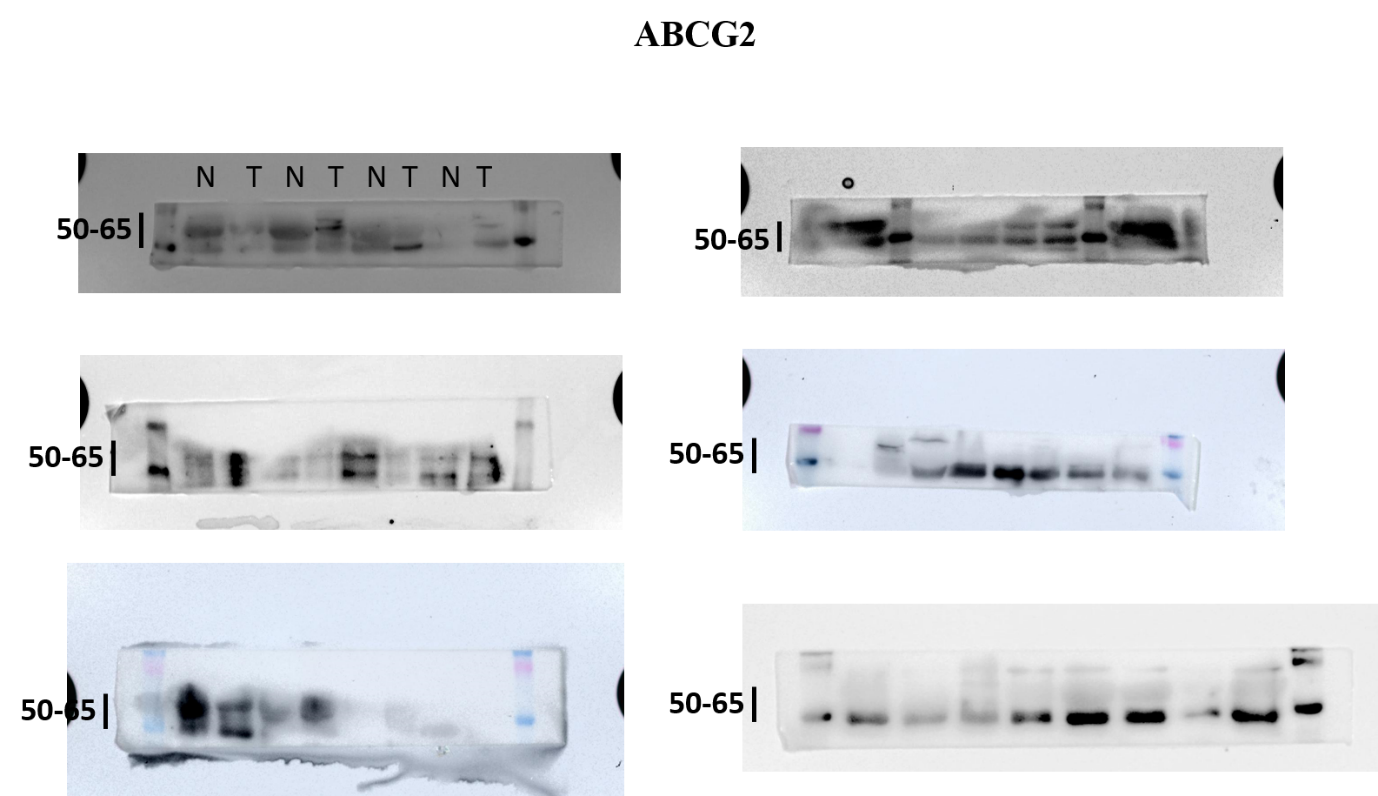


**Supplementary Figure 3.** Uncropped blot images showing ABCC1 and ABCG2


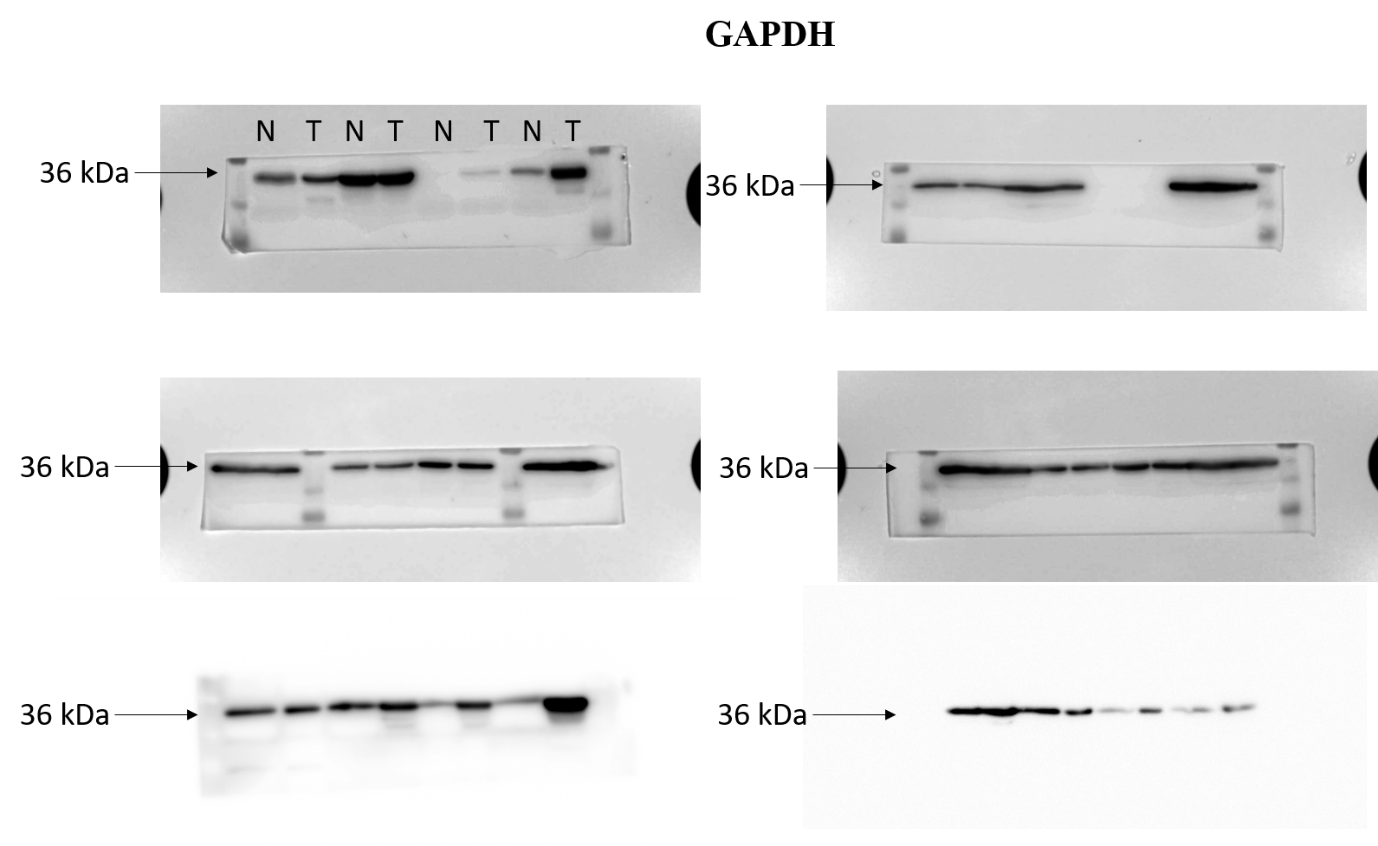


**Supplementary Figure 4.** Uncropped blot images showing GAPDH
